# Characterization of a Pathogen Induced Thaumatin-Like Protein Gene *AdTLP* from *Arachis diogoi*, a Wild Peanut

**DOI:** 10.1371/journal.pone.0083963

**Published:** 2013-12-19

**Authors:** Naveen Kumar Singh, Koppolu Raja Rajesh Kumar, Dilip Kumar, Pawan Shukla, P. B. Kirti

**Affiliations:** Department of Plant Sciences, University of Hyderabad, Hyderabad, India; Key Laboratory of Horticultural Plant Biology (MOE), China

## Abstract

Peanut (*Arachis hypogaea* L) is one of the widely cultivated and leading oilseed crops of the world and its yields are greatly affected by various biotic and abiotic stresses. *Arachis diogoi*, a wild relative of peanut, is an important source of genes for resistance against various stresses that affect peanut. In our previous study a thaumatin-like protein gene was found to be upregulated in a differential expression reverse transcription PCR (DDRT-PCR) study using the conidial spray of the late leaf spot pathogen, *Phaeoisariopsis personata*. In the present study, the corresponding full length cDNA was cloned using RACE-PCR and has been designated as *AdTLP*. It carried an open reading frame of 726 bp potentially capable of encoding a polypeptide of 241 amino acids with 16 conserved cysteine residues. The semi-quantitative RT-PCR analysis showed that the transcript level of *AdTLP* increased upon treatment with the late leaf spot pathogen of peanut, *P. personata* and various hormone treatments indicating its involvement in both, biotic and abiotic stresses. The antifungal activity of the purified recombinant protein was checked against different fungal pathogens, which showed enhanced anti-fungal activity compared to many other reported TLP proteins. The recombinant AdTLP-GFP fusion protein was found to be predominantly localized to extracellular spaces. Transgenic tobacco plants ectopically expressing *AdTLP* showed enhanced resistance to fungal pathogen, *Rhizoctonia solani*. The seedling assays showed enhanced tolerance of AdTLP transgenic plants against salt and oxidative stress. The transcript analysis of various defense related genes highlighted constitutively higher level expression of *PR1a*, *PI-I* and *PI-II* genes in transgenic plants. These results suggest that the *AdTLP* is a good candidate gene for enhancing stress resistance in crop plants.

## Introduction

In nature, plants are exposed to different kinds of environmental and biotic stresses such as attack by plant pathogens and insect predators. To defend themselves against these attacks, they have evolved various well established defense mechanisms. Induction of these mechanisms results in the activation of different stress related genes ultimately leading to the production of reactive oxygen species (ROS), accumulation of phytoalexins, hypersensitive response and synthesis of pathogenesis related (PR) proteins [1,2]. Van Loon et al. classified these PR proteins into 17 different families based on their structure, amino acid sequences and mode of action [3].

Members of PR-5 class proteins are also called thaumatin-like proteins (TLPs) because of their sequence similarity with the sweet tasting protein thaumatin from *Thaumatococcus danielli* [4]. TLPs are reported to be widely distributed PR proteins across kingdoms including gymnosperm, angiosperm, animal and fungal systems [5]. Molecular mass of TLPs ranges between 21 to 26 kDa with a thaumatin family signature G-X- [GF]-X-C-X-T- [GA]-D-C-X- (1,2)-G-X-(2,3)-C [6]. In general, TLPs have 16 conserved cysteine residues that are involved in the formation of eight disulfide linkages, which impart stability to the protein under varied thermal and pH conditions [7,8].

In vitro antifungal activity of TLPs has been reported against a variety of filamentous fungal pathogen such as *Botrytis cinerea, Fusarium oxysporum*, *Mycosphaerella arachidicola*, *Trichoderma viride* [9–11]. Osmotin like protein from black nightshade, *Solanum nigrum* also showed inhibitory effect on the growth of *Rhizoctonia batiticola* and *Sclerotinia sclerotiorum* [12]. TLPs possess the capacity to rupture the fungal membrane by pore formation [13]. In addition, they can also affect the fungus growth with β-glucanase and xylanase inhibitor activities [14,15]. Transgenic plants overexpressing TLP genes have shown enhanced resistance and protection against different fungal pathogens [16,17]. Some TLPs have also been reported to be involved in anti-freeze activity [18] and developmental processes like fruit ripening [19]. 

Peanut (*Arachis hypogaea* L), is an important legume/ oilseed crop cultivated worldwide and is very much susceptible to various biotic stresses caused by pathogens and insect pests resulting in huge losses in plant productivity. The germplasm belonging to the genus *Arachis* consists of 69 species with more than 40 wild relatives [20]. It has also been shown that these wild relatives exhibit high level of resistance/tolerance to the various stresses compared to the highly susceptible cultivated peanuts [20,21]. Hence, they are assumed to be an important source of genes for resistance to the stresses for peanut improvement. The leaf spots, collectively termed as ‘Tikka’ disease is a very serious disease affecting the cultivated peanuts. One of the pathogens responsible for Tikka disease is *Phaeoisariopsis personata* that causes late leaf spot; and early leaf spot is caused by *Cercospora arachidicola*. Wild peanut, *Arachis diogoi* exhibits very high levels of resistance to these and other diseases that seriously affect the cultivated peanuts. Through a differential display approach, we have earlier identified several genes that get significantly upregulated in *Arachis diogoi* upon treatment with *Phaeoisariopsis personata* [22]. One of the upregulate genes designated as AdDR-11 in the wild peanut was identified to encode a putative thaumatin-like protein [22]. 

In the present study, using the available partial cDNA sequence of AdDR-11, full length cDNA was amplified and cloned from *Arachis diogoi* and it was named as *AdTLP*. We analyzed its transcriptional regulation in response to various treatments and its subcellular localization by translational fusion with GFP. *In vitro* and *in vivo* antifungal activity of AdTLP protein was analyzed against different fungal pathogens, *Botrytis cinerea*, *Fusarium oxysporum*, *Fusarium solani and Rhizoctonia solani*. Abiotic stress assays were also carried out to observe the efficacy of AdTLP in alleviating stress caused high NaCl and H_2_O_2_ conditions. In addition to this, transcript levels of various defense responsive genes were checked in transgenic plants and our results are reported in this communication.

## Materials and Methods

### Plant materials and treatments

Wild peanut (*Arachis diogoi*) and tobacco (*Nicotiana tabacum* var Xanthi) plants were maintained in the green house. For different treatments, detached leaves of *A. diogoi* were utilized and the experiments were performed essentially as described earlier [22]. In brief, 10^5^ conidia per milliliter of the peanut late leaf spot pathogen, *Phaeoisariopsis personata* were used for pathogen treatment. For hormone treatments, concentrations of 500 μM salicylic acid (SA), 100 μM methyl jasmonate (MeJA), 100 μM abscisic acid (ABA), and 250 μM ethephon were utilized. Samples were collected at regular intervals, quick-frozen in liquid nitrogen, and stored at -80° C till further use.

### 5′ RACE, Isolation of Full Length cDNA and Genomic Sequence of *AdTLP*

RNA was isolated from frozen samples using RNeasy Plant Mini Kit (Qiagen, Germany). Isolated RNA was quantified and the quality was evaluated through formaldehyde gel electrophoresis. Rapid amplification of cDNA ends (RACE) was performed using 5′/3′ RACE kit (Roche Applied Sciences, Germany) following the manufacturer’s instructions. In brief, two micrograms of total RNA was reverse transcribed with gene specific primer AdDR11-146R using Transcriptor reverse transcriptase provided in the kit. The dA-tail was added to the synthesized first strand cDNA at the 5′ end using terminal transferase. The dA-tailed cDNA was used as template in subsequent PCR reactions. Gene specific primers AdDR11-117R and AdDR11-64R were designed based on the available partial sequence (AdDR-11) [22]. It was used in combination with Oligo-dT anchor primer and PCR anchor primer in the PCR reactions respectively. RACE-PCR reactions were performed using *Taq* DNA polymerase (Sigma-Aldrich). Genomic DNA served as templates for the amplification of genomic sequences. All PCR products described in the study were cloned in to pTZ57R vector (Fermentas, Germany) and sequenced commercially for sequence confirmation. All the primer details were provided in Table 1.

**Table 1:** Sequences of the oligonucleotides used in the study (see text for details).

**Name of the primers**	**Primer sequences (5’-3’)**
AdDR11-64R	CATTAGGGCACTGGTTGCTA
AdDR11-117R	GCCTCCTGAACAAGTGAAAG
AdDR11-146R	CATGGACAGAAGTTGATAGC
TLP ORF-F	GGGATCCATGGCGATTACTCGTGTTGT
TLP ORF-R	CCTCGAGTCATGGACAGAAGTTGATAGC
TLP ORF-F1	GGGGCCCATGGCGATTACTCGTGTTGT
TLP ORF-R1	GGGATCCTCATGGACAGAAGTTGATAGC
TLP ORF-F2	GGGGCCCATGGCGATTACTCGTGTTGT
TLP ORF-R2	CCCCGGGTCATGGACAGAAGTTGATAGC
PCR anchor primer	GACCACGCGTATCGATGTCGAC
Oligo dT-anchor primer	GACCACGCGTATCGATGTCGACTTTTTTTTTTTTTTTTV
Actin-F	TGGCATCACACTTTCTACAA
Actin-R	CAACGGAATCTCTCAGCTCC

### Analysis of cDNA and protein sequence

The cDNA sequence data were analyzed using BLASTn and BLASTp at NCBI website (http://www.ncbi.nlm.nih.gov). The theoretical isoelectric point and molecular mass were computed using COMPUE pI/Mw tool (http://web.expasy.org/compute_pi/). Nucleotide translations were performed using translate tool at ExPASy (http://www.expasy.ch/). Signal peptides were predicted using SignalP 4.1 (http://www.cbs.dtu.dk/services/SignalP/). Multiple sequence alignment and phylogenetic tree were done using ClustalW (www.ebi.ac.uk) and MEGA 4.0.2 software respectively.

### Semi-quantitative RT-PCR

First strand cDNA was synthesized by reverse transcribing RNA (500ng) using MMLV reverse transcriptase (Sigma-Aldrich). Subsequent PCR reactions were performed using the first strand cDNA as template in the presence of gene specific primers ORF-F and ORF-R. PCR reactions were standardized empirically to keep the amplification in linear range. For all PCR reactions *Taq* DNA polymerase (Sigma-Aldrich) was used.

### AdTLP subcellular localization

The ORF of *AdTLP* was amplified using gene specific primers ORF-F1 and ORF-F2 harboring *Apa*I and *Bam*HI restriction sites respectively with a proofreading polymerase. The amplification product was digested with *Apa*I and *Bam*HI and cloned into pRT-GFP vector [22] using the same set of enzymes to generate 35S:AdTLP:GFP. The AdTLP:GFP expression cassette was released using *Sph*I and further sub-cloned into *Hin*dIII site of binary vector pCAMBIA1300 after end filling both the sites, which resulted in AdTLP:GFP:1300. The recombinant binary vector was mobilized into *Agrobacterium* strain LBA4404 using freeze thaw method. Similarly empty vector pEGAD for the expression of free GFP was also mobilized into the *Agrobacterium* strain.

### Agroinfiltration and microscopy

Agroinfiltration was performed essentially as described earlier [22]. In brief, agrobacterial strains harboring the free GFP and recombinant AdTLP-GFP vectors were grown in LB medium in the presence of appropriate antibiotics. The agrobacterial cultures were pelleted and resuspended in the infiltration medium (10mM MES, 10mM MgCl_2_ and 200µM acetosyringone) adjusted to an OD_600_ of 0.8. Agrobacterial suspensions were then infiltrated into the adaxial side of the tobacco leaves using a needless syringe. GFP expression was visualized in infiltrated leaves 48-96 hours post infiltration (hpi) at different time intervals with laser scanning confocal microscopy (Leica TCS SP2 with Leica DM6000 microscope).

### Expression and purification of AdTLP protein

 The 726 bp open reading frame was amplified using ORF-F and ORF-R primers with *Bam*HI and *Xho*I sites, digested and cloned into the corresponding sites of pET32a(+) expression vector (Novagen Corporation, USA). The cloned vector was transformed into cells of *E.coli* Rosetta gami-2 (DE3) strain and the transformed cells were grown at 37° C in Luria and Bertani (LB) broth (Himedia, India) with antibiotics chloramphenicol (25 µg ml^-1^) and ampicillin (100 µg ml^-1^) overnight at 200 rpm in an orbital shaker. A 10 mL aliquot from the overnight grown culture was added to 1L of fresh LB medium and grown further at 37° C until the OD_600_ reached 0.5-0.6. Then, these cells were induced to express recombinant protein by adding 1mM IPTG (Fermentas, Germany) for 4 h. Following the induction, the induced cells were harvested by centrifugation at 5000 rpm for 10 min at 4° C. Recombinant proteins were exclusively found in inclusion bodies, which was confirmed by resuspending 2 mL culture pellet in lysis buffer ( 200 mM Tris-cl pH 7.5, 10mM EDTA, 1% Triton X-100) followed by sonication and SDS-PAGE analysis. To purify the protein from inclusion bodies, pelleted cells from 1 L culture were resuspended in lysis buffer (8 M urea, 10mM imidazole, 0.1M NaHPO_4,_ 0.01M Tris base pH 8) and sonicated. The lysate was centrifuged at 12,000 rpm for 20 min to remove the debris. The expression of the fusion protein with the N-terminal fusion partners, Thioredoxin-His-S-tag linked to the target peptide (AdTLP) and purification was done by IMAC (immobilized metal ion affinity chromatography) using the His-Tag of the fusion protein. Supernatant was mixed with 2 mL of equilibrated Nickel-NTA resin and added into the column. The flow through was collected and wash it with 10 volumes of wash buffer (8 M urea, 50mM imidazole, 0.1M NaHPO4, 0.01M Tris base, pH 6.3) and finally the recombinant peptide was collected with elution buffer (8 M urea, 250 mM imidazole, 0.1M NaHPO4, 0.01 M Tris base, pH 4.5). Dialysis was conducted for protein renaturation by gradually adding renaturation buffer (Urea, 0.1 M NaH_2_PO_4,_ 0.01 M Tris base, pH 7.0) containing decreasing concentration of urea from 6M to 1 mM. Then Tris buffer (0.01 M) alone was used for five times with an interval of 1 h. Finally it was kept for overnight dialysis in the same buffer at 4° C. The purified protein was resolved in 12% SDS-PAGE. Protein concentration was measured using Bradford method.

### Antifungal activity assay

Antifungal activity of the recombinant AdTLP (rAdTLP) protein was tested by microspectrophotometry as well as in vitro plate assay with the fungal pathogens, *Fusarium oxysporum, Fusarium solani, Botrytis cinerea, Rhizoctonia solani*. For microspectrometry analysis, standard method as described by Song et al. [23] and Vijayan et al. [24] was followed. Briefly, 10µl of protein, diluted to different concentrations, was pipette into the wells of a 96-well microtiter plate containing 140µl of test fungal spore suspension (~ 3.0× 10^4^ spores/ mL) in potato dextrose broth, which was placed in an incubator at 28° C. Antifungal activity of each concentration of protein was performed in triplicates. Fungal spore germination was observed microscopically, whereas optical density at 595 nm wavelength was measured to check the spore growth after inoculation for 30 min and 48 hrs. Controls that were devoid of the test protein were tested for comparing the antifungal activity of the rAdTLP. Values of growth inhibition less than 10% were not considered as significant. Growth inhibition is defined as the ratio of the corrected absorbance at 595 nm of the control minus the corrected absorbance of the test sample, divided by the corrected absorbance of the control. The corrected absorbance is defined as the absorbance at 48 h minus that at 30 min. IC_50_ is defined as the protein concentration at which 50% inhibition was reached [24].

For the *in vitro* plate assay, fungal discs of uniform size were inoculated at the centre of the Potato Dextrose agar media and incubated at 28° C. When the mycelial spread reached 4 cm in diameter, four sterile Whatman no.1 filter paper discs of equal size were placed at equal distance from centre. Purified protein was added at various concentrations (ranging from 10-50 µg/ mL) at the centre of disc on the plate. The elution buffer served as control and the plates were incubated at 28° C. Growth of mycelia was observed periodically till they covered the control discs. A graph was plotted showing percentage inhibition of fungal growth against the concentration of protein to determine the IC_50_ for *Rhizoctonia solani*.

### β-1, 3 Glucanase assay

β-1, 3 Glucanase assay was done using the method of Looze et al. [25] with some modifications. The activity of rAdTLP proteins was analyzed by mixing 50 µg of protein sample with 100 µL of 50 mM acetate buffer (pH 5.0) containing 1% Laminarin (Sigma). The mixture was incubated at 37° C for different time periods ranging from 30 min to 3 days. Absorbance was measured at 595 nm. Increase in absorbance indicates β-1, 3 glucanase activity.

### Agrobacterium mediated Tobacco transformation with 35s-AdTLP construct

The *AdTLP* ORF was reamplified using ORF-F2 and ORF-R2 primers having *Apa*I and *Sma*I sites respectively, digested and cloned in pRT100 vector. Due to presence of internal sites, partial digestion was performed with *Hin*dIII enzyme for releasing ~1.5 Kb cassette containing the CaMV35S promoter and polyadenylation signal from pRT100 vector along with the *AdTLP*. The *AdTLP* expression cassette was further cloned in the binary vector pCAMBIA2300 at the *Hin*dIII site in the multiple cloning sites. Tobacco (*Nicotiana tabacum* cv Xanthi) was transformed through leaf disc transformation using *Agrobacterium tumefaciens* strain EHA105 carrying the binary vector pCAMBIA2300-AdTLP and the transformants were selected on 125mgL^-1^ kanamycin [26]. T_0_ putative transgenic were analyzed by polymerase chain reaction (PCR) and reverse transcriptase polymerase chain reaction (RT-PCR) for the target gene. Among seven different transgenic plants analyzed, progenies of the primary transgenic plants 4 (low expression *AdTLP* plant) and #7 (high expression *AdTLP* plant) were taken for further analysis T_1_ and T_2_ seeds were raised via self-pollination and T_2_ seeds were used in functional characterization.

### Evaluation of transgenic plants for resistance against fungal pathogen

Root bioassay, using the fungal pathogen, *Rhizoctonia solani*, was carried out to check the resistance in the transgenic plants expressing *AdTLP* constitutively against the root rot causing fungal pathogen. At maturity, seeds from transgenic plants were collected and germinated for T_2_ generation analysis. Seeds from line 4 and 7 were surface sterilized and grown on half strength MS medium (MSH). After germination, they were transferred to the cups filled with sterilized vermiculite and soil in 3:1 ratio. Four seedlings were transferred to each cup and allowed to grow further for another 25 days. Control seedlings were transferred simultaneously. Ten sclerotia of equal size were added to each cup and they were maintained under humid condition by covering with polyethylene covers in the growth room. Symptoms started appearing after 5 days and observations were taken after 8 days and 10 days of post infection (dpi). 

### Abiotic stress assays

Seedlings of transgenic and wild plants (WT) were selected to analyse the salt (sodium chloride) and oxidative stress tolerance. T_2_ transgenic seedlings were grown on half MSH medium (without organics) containing 125mgL^-1^ kanamycin and simultaneously, the WT plants were grown on MSH medium without kanamycin. Twenty seedlings each of WT and kanamycin resistant seedling in T_2_ generation (plants 4 and 7) were transferred to different stress treatment plates for each experiment. For salt stress seedlings were transferred to 100 mM, 200 mM and 300 mM NaCl in MSH. For oxidative stress treatment 2% H_2_O_2_ in MSH medium was used. Total chlorophyll content was measured spectrophotometrically as described by Arnon [27]. Lipid peroxidation was assessed by measuring thiobarbituric acid reactive substances (TBARS) as described by Heath and Packer [28]. In the case of NaCl treatment, both the chlorophyll and TBARS estimation were done after 12 days of treatment and for H_2_O_2,_ after 10 days of treatment. 

### Expression analysis of other stress related genes

 Transcript accumulation for some defense responsive genes was monitored in WT and transgenic plants, using semi quantitative RT-PCR. Leaf samples were collected from two months old plants, quick frozen and stored in -80° C. Primer sequences used in this study were provided in Table S1.

## Results

### Isolation of full length cDNA, genomic sequence of *AdTLP* and their analysis

**Figure 1:**
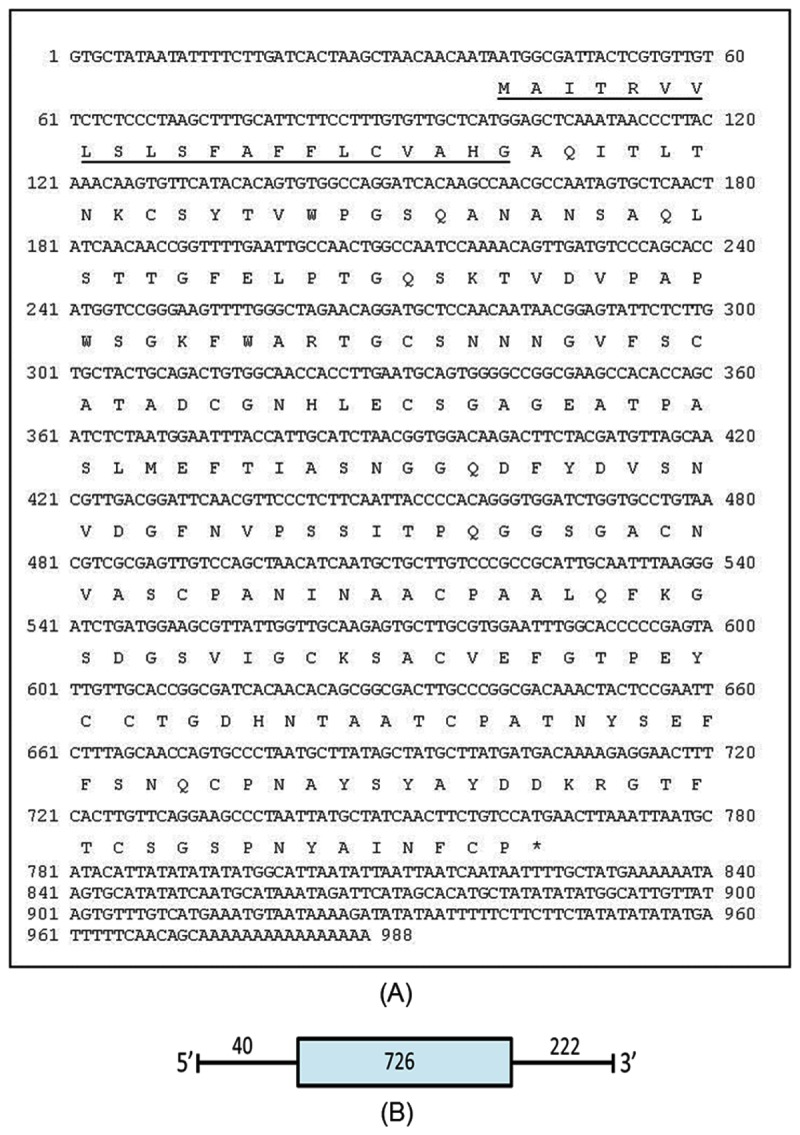
The nucleotide, deduced amino acid sequence and gene structure of *AdTLP*. Deduced amino acid sequence of the protein is shown under the nucleic acid sequence (A). Nucleotides are numbered. (*) indicates a stop codon. A 21 amino acid N-terminal signal peptide is underlined. The gene structure of *AdTLP* is shown in Figure (B). Exon is represented by closed box and dark lines represent 5′ and 3′ UTRs. Their respective lengths were given in bp.

A partial cDNA clone of 369bp designated as AdDR-11 encoding a putative thaumatin-like protein was isolated in a previous study [22]. Using 5' RACE-PCR approach, a 686bp product was obtained using gene specific primer AdDR11-64R and PCR anchor primer. Full length cDNA sequence was deduced based on the overlapping sequence of the obtained RACE product with existing partial cDNA sequence. The full length cDNA was amplified and confirmed after sequencing. It showed homology with thaumatin-like proteins and was hence, designated as *AdTLP*, and the sequence was deposited in the NCBI Genbank under the accession number FJ481982. The cDNA was 988 bp in length with an open reading frame of 726 bp potentially encoding a polypeptide of 241 amino acids (Figure 1A). The theoritical *pI* and the molecular mass of *AdTLP* were 4.71 and 25005.63 Da respectively including the signal peptide. An N-terminal signal peptide of 21 amino acids was present suggesting it to be a secretory protein (Figure 1A). The amplification of corresponding genomic sequence revealed that *AdTLP* gene did not harbor any introns (Figure 1B).

**Figure 2:**
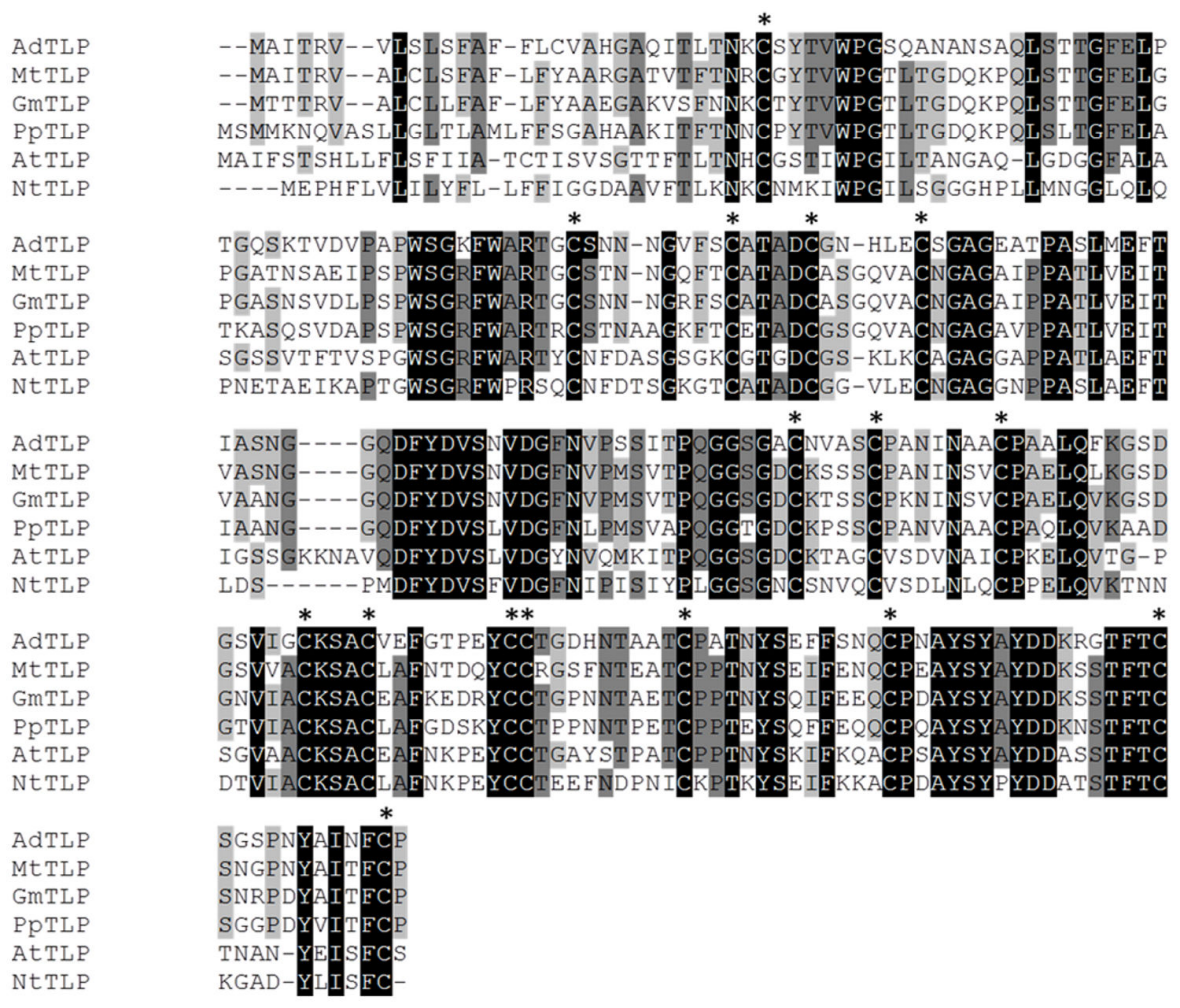
Multiple sequence alignment of AdTLP with TLPs from other plant species. The sixteen cysteine residues required for the formation of eight disulfide bridges are conserved in *AdTLP* also and are indicated by asterisk (*). Mt: *Medicago truncatula*, Gm: *Gycine* *max*, Pp: *Pyrus pyrifolia*, At: *Arabidopsis thaliana*, Nt: *Nicotiana tabacum*.

**Figure 3:**
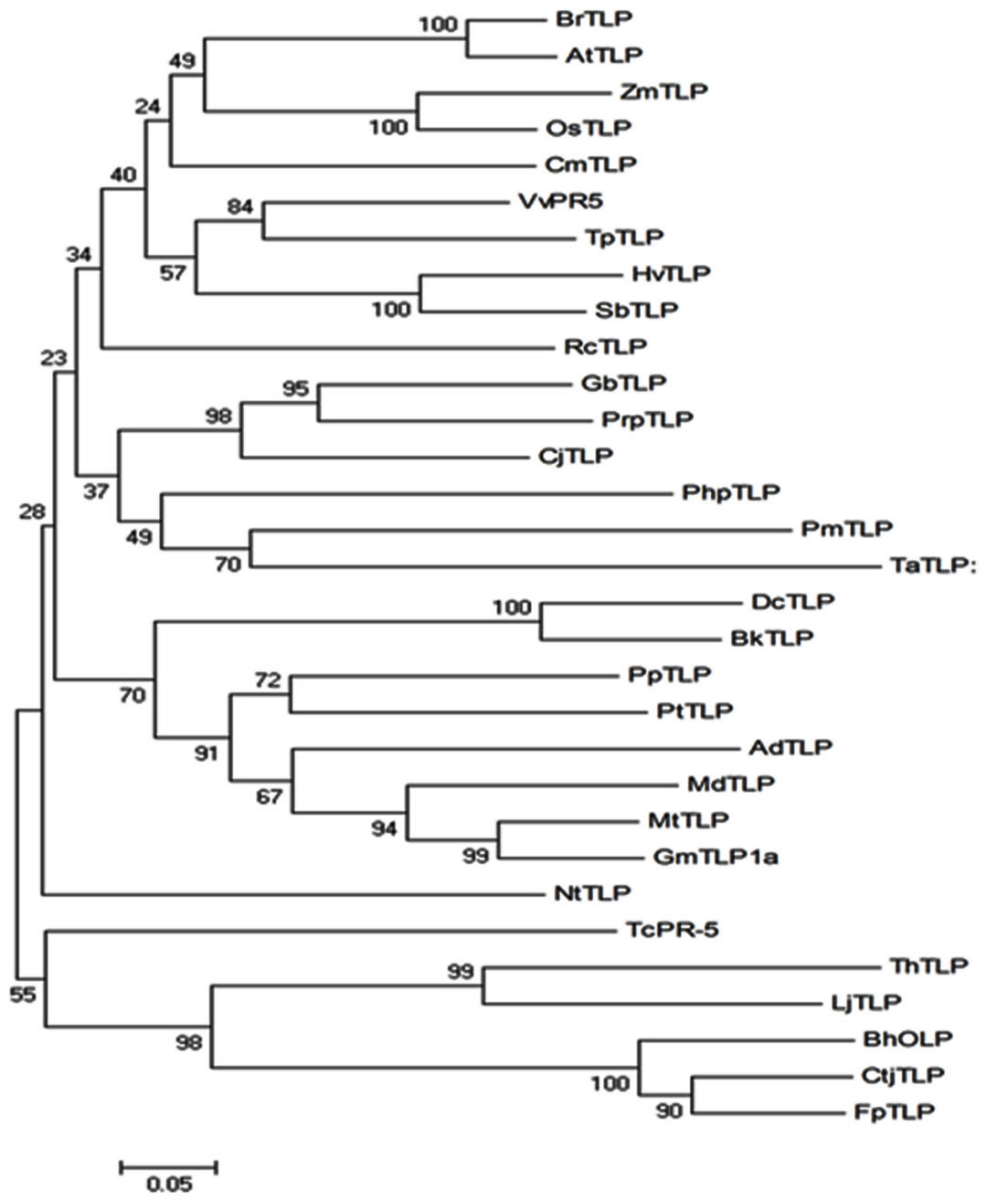
Phylogenetic analysis of *AdTLP* with other TLPs. A phylogenetic tree based on genetic distance of the protein sequences was constructed using MEGA 4.0.2 software. Bootstrap values are indicated at the branches. The TLP members used for construction of the tree are listed in the GenBank database under the following accession numbers: *AtTLP* (AAD02499.1)*; BhOLP* (AAD53089.1); *BkTLP* (CBJ55937.1); *BrTLP* (ABV89616.1); *CjTLP* (BAD90814.1); *CmTLP* (ADN33945.1); *CtjTLP* (BAI63297.1); *DcTLP* (AAL47574.1); *FpTLP* (ABB86299.1); *GbTLP* (ABL86687.1); *GmTLP1a* (XP_003535214.1); *HvTLP* (BAJ96850.1); *LjTLP* (AFK33451.1); *MdTLP* (AAC36740.1); *MtTLP* (AFK34461.1); *NtTLP* (BAA74546.2); *OsTLP* (BAD34224.1); *PhpTLP* (XP_001784610.1); *PpTLP* (BAC78212.1); *PmTLP* (ADB97928.1); *PrpTLP* (AEV57470.1); *PtTLP* (XP_002330973.1); *RcTLP* (XP_002519620.1); *SbTLP* (XP_002465570.1); *TaTLP* (AAM15877.1); *ThTLP* (BAJ34394.1); *TpTLP* (BAE71242.1); *VvPR5* (XP_002277548.1); *ZmTLP* (NP_001142502.1); At *Arabidopsis thaliana, Bh* *Benincasa hispida*, Bk *Bupleurum kaoi*, Br *Brassica rapa, Cj* *Cryptomeria japonica, Cm Cucumis melo, Ctj* *Citrus jambhiri, Dc* *Daucus carota, Fp Ficus pumila, Gb* *Gossypium barbadense, Gm* *Glycine max, Hv* *Hordeum vulgare, Lj* *Lotus japonicas*, Md *Malus domestica*, Mt *Medicago truncatula, Nt* *Nicotiana tabacum*, Os *Oryza sativa, Php* *Physcomitrella patens, Pp* *Pyrus pyrifolia, Pm Pinus monticola, Prp* *Prunus persica*, Pt *Populus trichocarp, Rc Ricinus communis, Sb* *Sorghum bicolor*, Ta *Triticum aestivum*, Th *Thellungiella halophile, Tp Trifolium pratense, Vv Vitis vinifera, Zm Zea mays*.

Sequence alignment of deduced amino acid AdTLP with TLPs from other plants showed that there were sixteen cysteine residues involved in formation of eight disulfide bridges and are conserved across the species. The AdTLP protein exhibited 68% sequence similarity with MtTLP from *Medicago trancatula*, 66% to GmTLP from *Glycine max*, and 62% to PpTLP from *Pyrus pyrifolia* resepectively. Among well characterized TLPs, *Arabidopsis thaliana* TLP (AtTLP) and *Nicotiana tabacum* TLP (NtTLP) shared 50% and 47% similarity with AdTLP respectively (Figure 2). Phylogenetic analysis (Figure 3) showed that the AdTLP was closely related to a non-leguminous MdTLP (*Malus domestica*), as well as leguminous MtTLP (*Medicago trancatula*) and GmTLP (*Glycine max*).

### Transcript Expression Analysis

**Figure 4:**
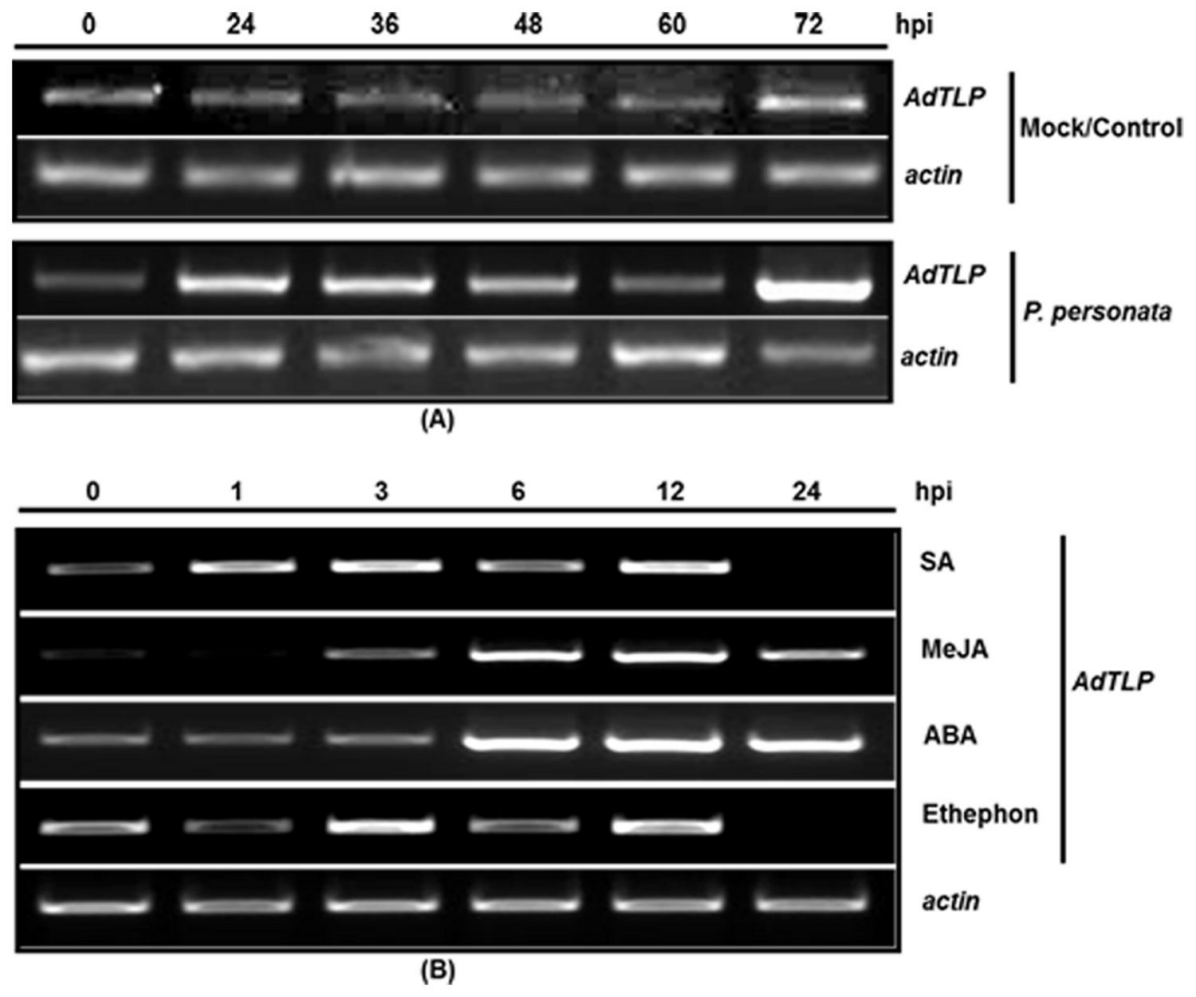
Transcript level analysis of *AdTLP* in *A. diogoi*. Transcript level of *AdTLP* in *A. diogoi* was analyzed using semi-quantitative RT-PCR, during *P. personata* (A) and various hormones treatments (B).

Transcript levels of *AdTLP* were analyzed using semi-quantitative RT-PCR in response to pathogen infections and different stress hormones. *AdTLP* ORF-F and ORF-R primers were used to amplify *AdTLP*. During the pathogen infection, upregulation of *AdTLP* was observed as early as 24 hpi, which gradually reached the basal level by 48 hpi and again rebounding to a very high level by 72 hpi (Figure 4A). Early upregulation of transcripts were observed during SA treatment, which was persistent till 12 hpi whereas they got upregulated at later stages during JA and ABA treatment (Figure 4B).

### Localization of AdTLP

**Figure 5:**
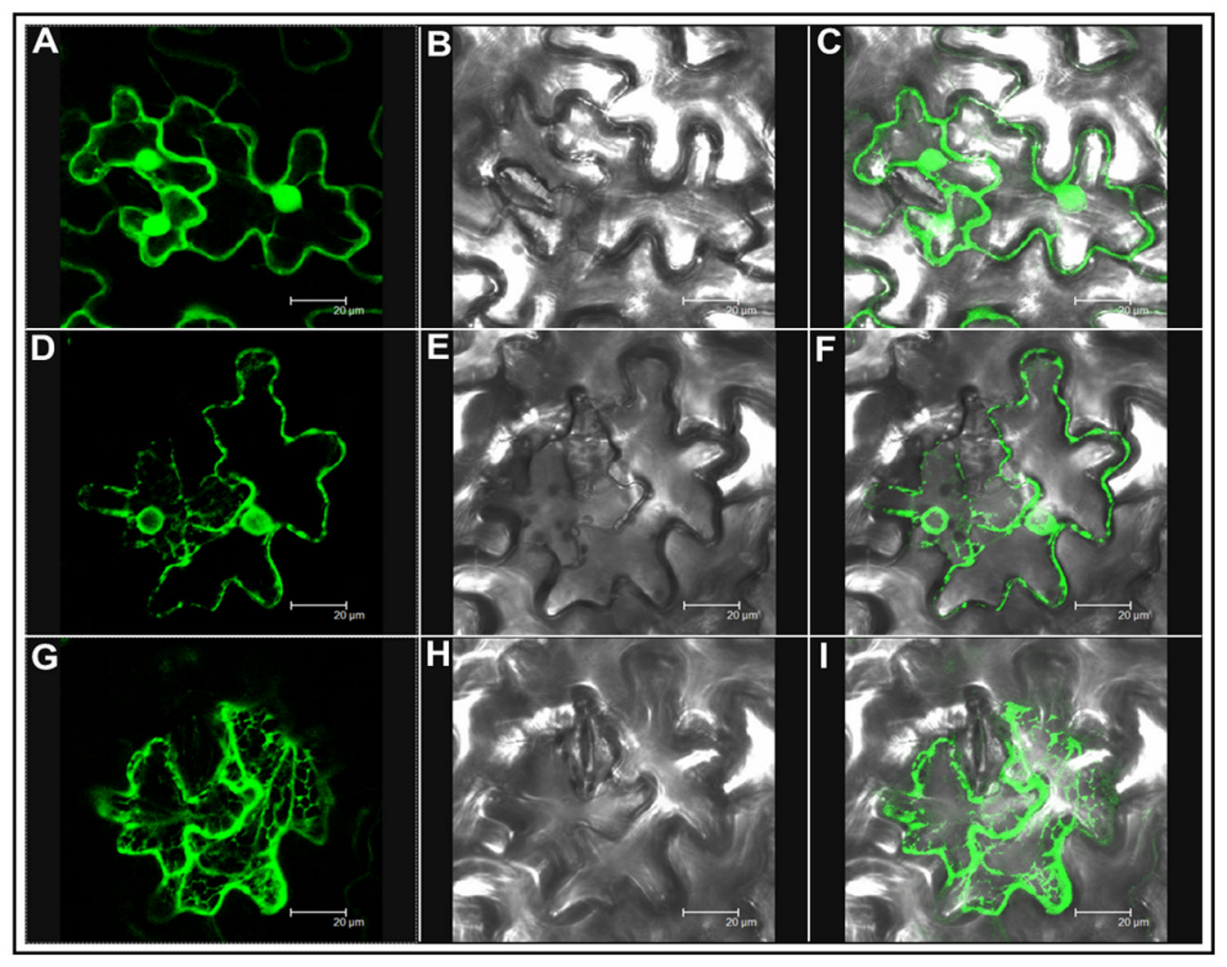
Subcellular localization of *AdTLP* by transient expression in tobacco leaves using agroinfiltration. Empty vector pEGAD expressing free GFP (A, B, C). AdTLP:GFP:1300 expressing translationally fused AdTLP-GFP and cells were visualized at different planes (D-I). Expression of free GFP and AdTLP-GFP in epidermal cells (A, D, G), corresponding bright field image (B, E, H), and overlay of GFP signal onto bright field image (C, F, I).

To study the subcellular distribution of AdTLP, a C-terminal translational fusion with GFP was constructed. When expressed transiently in the epidermal cells of tobacco leaves, the free GFP was found to be accumulated in both cytosol and nucleus (Figure 5A). The recombinant AdTLP-GFP protein exhibited predominant localization in extracellular spaces with some presence in nuclear boundaries as well (Figure 5D). When the AdTLP-GFP expression cells were visualised at different planes, the distribution of AdTLP-GFP was also observed in subcellular structures, possibly endoplasmic reticulum (Figure 5G).

### Prokaryotic expression and purification

**Figure 6:**
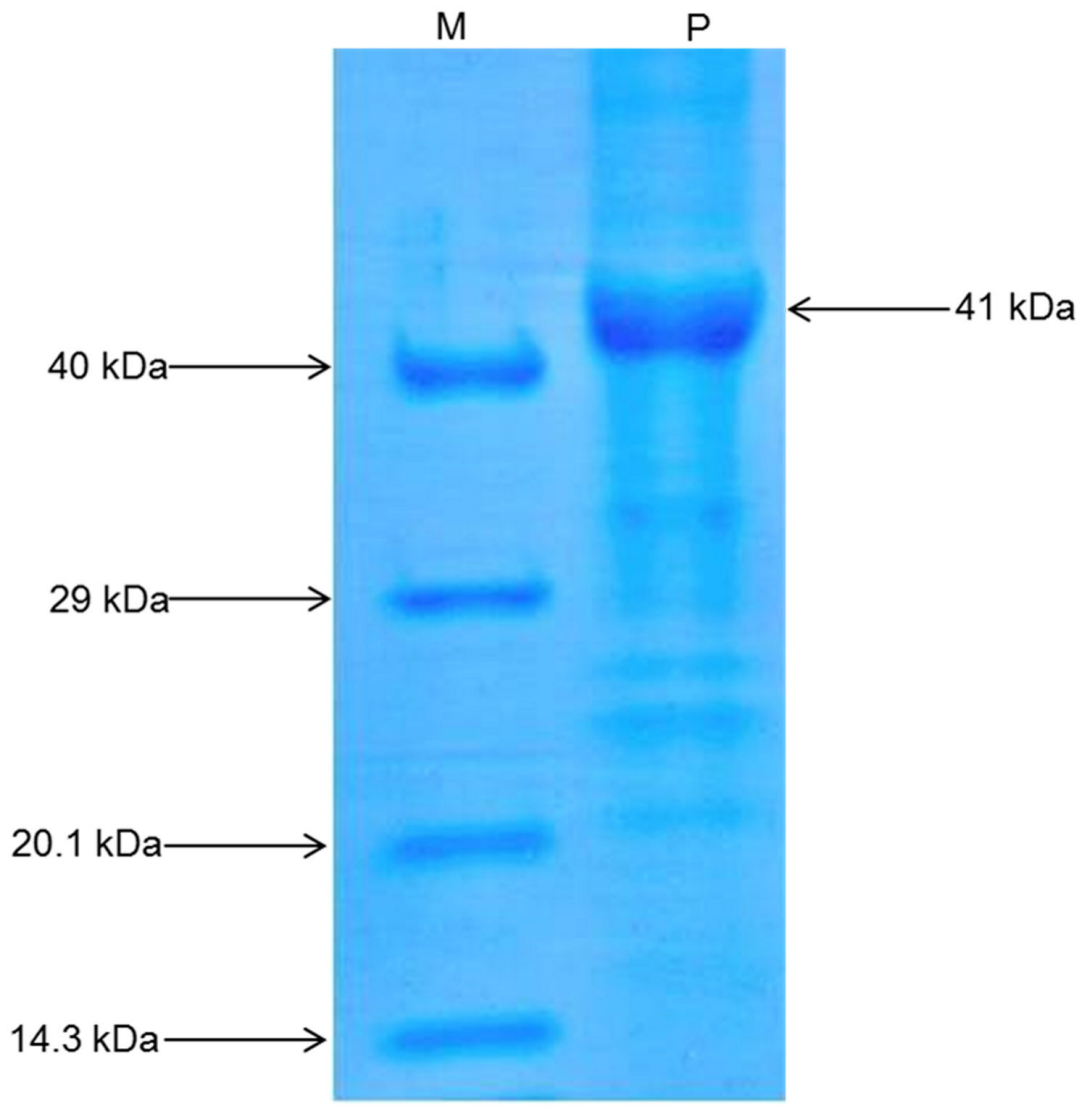
SDS-PAGE analysis of purified protein. 12% SDS-PAGE analysis showing the purified protein. (M) Protein marker, (P) purified recombinant AdTLP protein.

The *AdTLP* is supposed to code for a protein with 241 amino acids with a protein mass of ~25 kDa. The recombinant protein of 41 kDa, including 16 kDa tag region of pET32a vector, was expressed upon induction with 1mM IPTG (Figure S1). The protein was exclusively found in insoluble fraction in the inclusion bodies. Low temperature treatment and various concentration of IPTG did not show any effect on protein solubility. The recombinant protein was isolated from the inclusion bodies and solubilised using a buffer containing urea. Further it was refolded by using dialysis. This purified protein was used for *in vitro* fungal assay against different fungal pathogens (Figure 6).

### In vitro antifungal activity and endo β-1, 3 Glucanase assay

**Figure 7:**
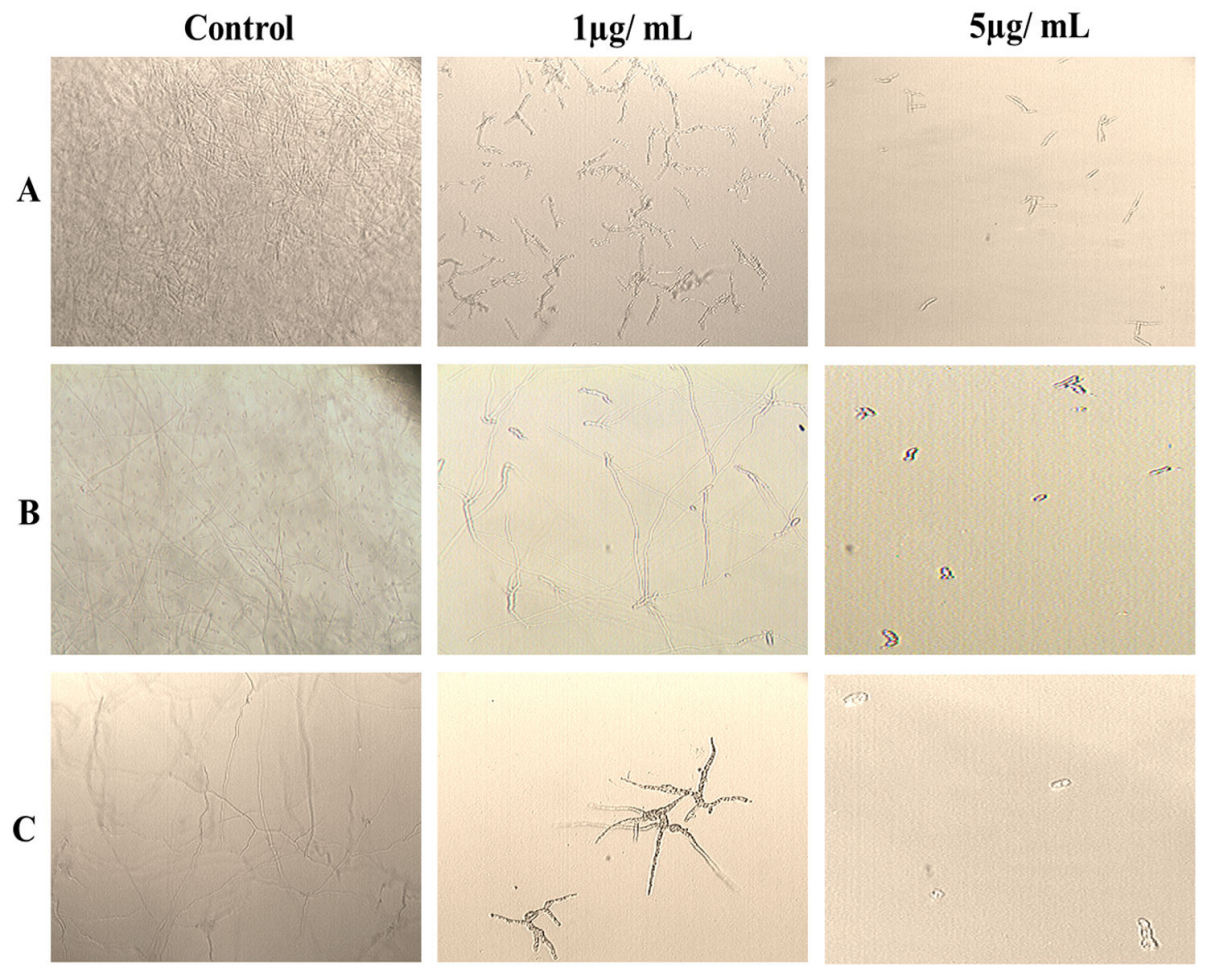
Fungal spore germination assay in the presence of different concentrations of AdTLP protein. A. *Fusarium oxysporum* B. *Fusarium* *solani* C. *Botrytis cinerea*.

Antifungal activity of *AdTLP* protein was investigated using spore germination and plate assays. During spore germination assays with *F. oxysporum, F. solani* and *B. cinerea*, drastic decrease in hyphal growth was observed even in protein concentration as low as 1 µg/ mL of the recombinant protein (Figure 7). For these three fungal species, the calculated IC_50_ values were less than 1 µg/ mL. Hyperbranching of mycelium was also observed, which was very much distinct in the case of *B. cinerea*. A 5µg/ mL concentration of proteins was sufficient to stop the fungal spore germination completely.

**Figure 8:**
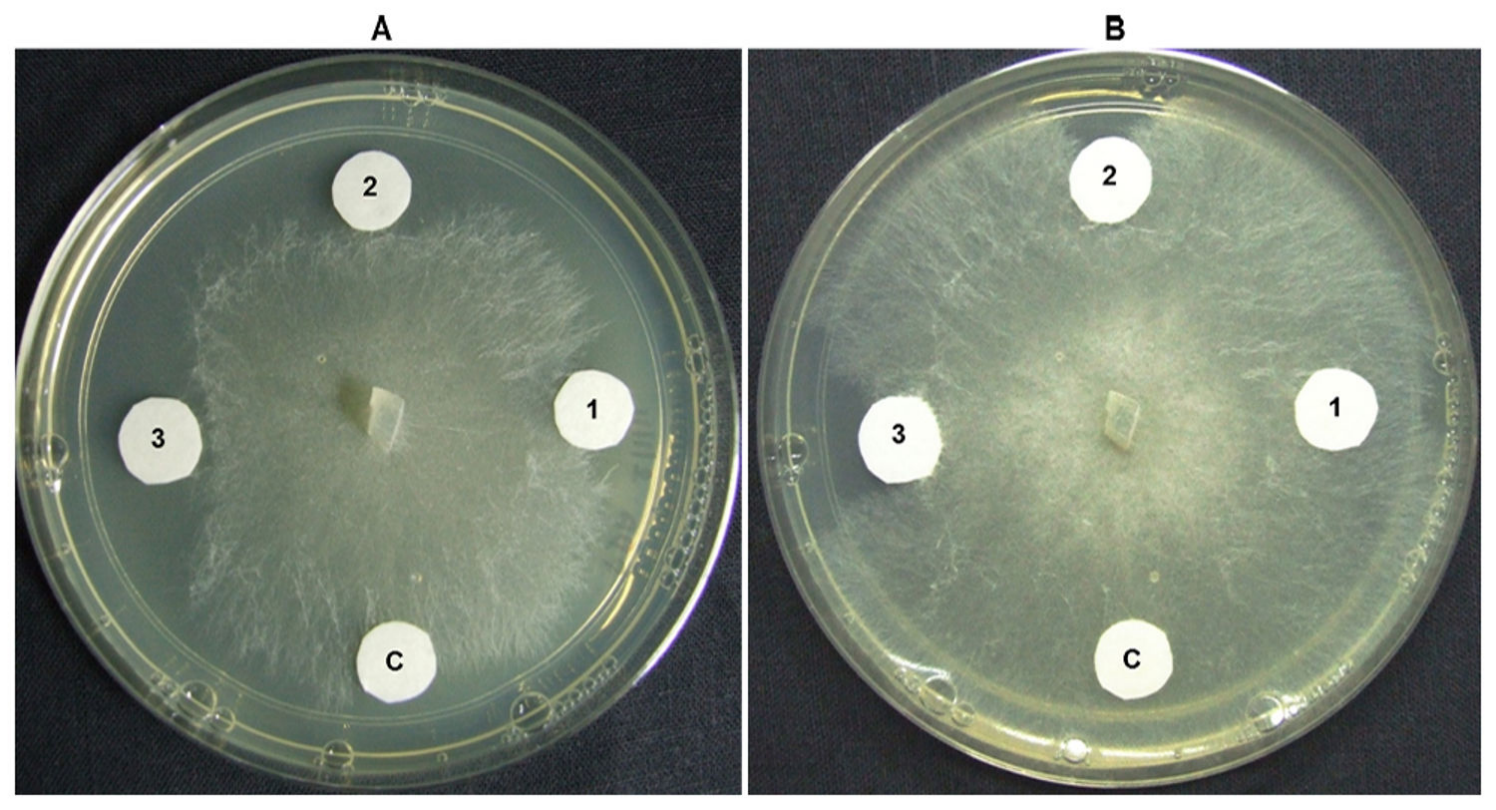
Effect of recombinant AdTLP protein on the growth of *Rhizoctonia* *solani*. C, 1, 2 & 3 represents control, 10µg/ mL, 25µg/ mL, and 50µg/ mL, respectively. Photographs were taken after 24 and 36 hrs of fungal growth (A and B).

Plate assay of protein was also performed with *Rhizoctonia solani*. There were varied zones of inhibition in the test fungus depending on the protein concentration applied. A graph was plotted between percentage growth inhibition and protein concentration (data not shown) and the IC_50_ value was calculated to be 38 µg/ mL for this pathogen (Figure 8).

To observe whether AdTLP exhibits any β-1, 3 glucanase activity, 50 µg of recombinant protein was incubated with Laminarin for 30 min to 3 d. No detectable absorbance was observed at 595nm indicating that AdTLP did not possess any identifiable glucanase activity.

### Genomic DNA PCR and RT-PCR analysis on putative transgenic plants

**Figure 9:**
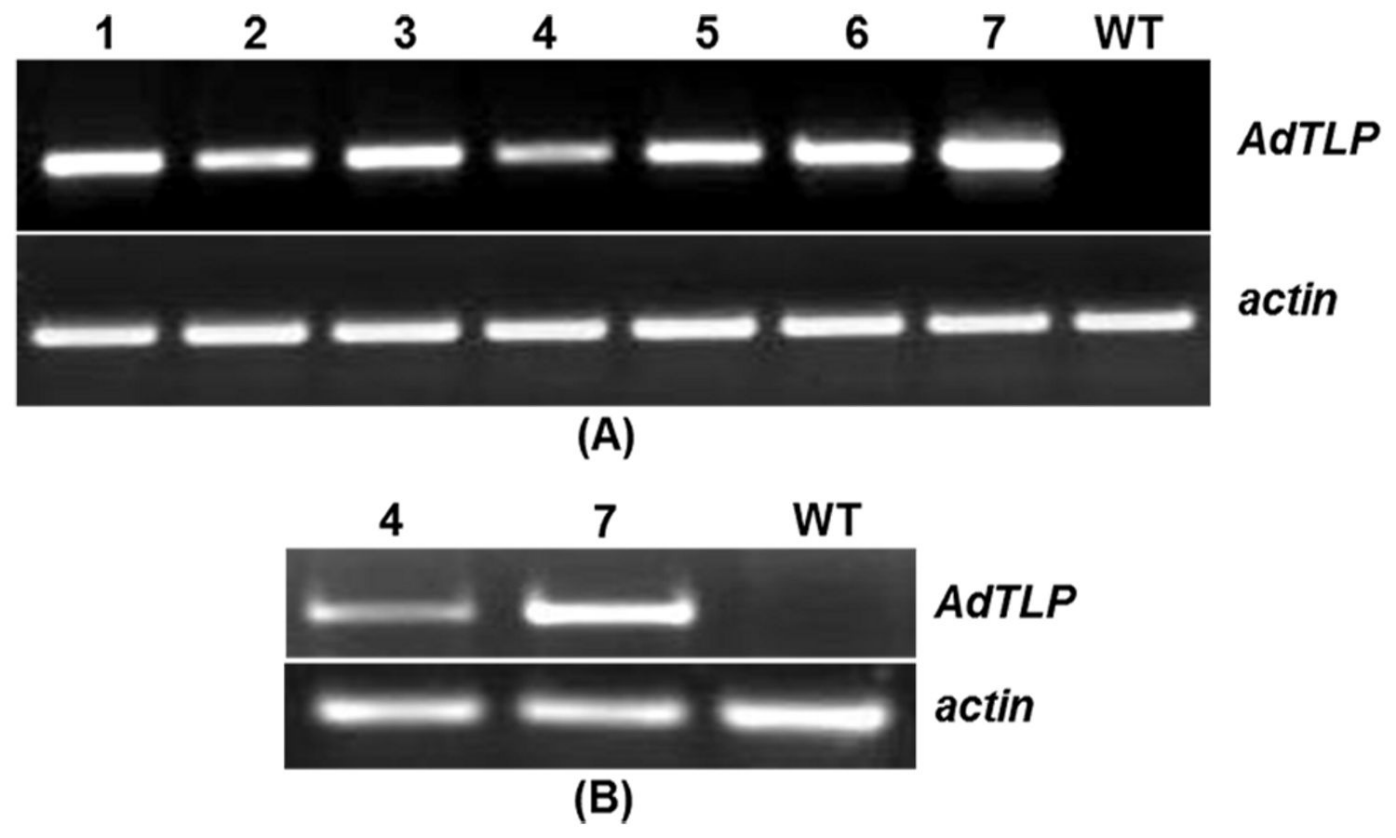
Semi-quantitative RT-PCR analysis of transgenic plants. Transcript levels of *AdTLP* were checked in T_0_ (A) and T_2_ (B) transgenic plants. Line 7 is high expression line and line 4 represents low expression line. Actin served as control to demonstrate equal loading.

To identify the primary transgenic plants with high and low level expression of the target gene, *AdTLP*, RT-PCR analysis was performed. Genomic DNA was isolated from ten different kanamycin positive plants and PCR reaction was performed by using primers for the marker genes, *npt*II marker and *AdTLP*. Out of ten plants analyzed, seven plants gave expected amplification of 700 bp and 726 bp fragments respectively for *npt*II and *AdTLP* sequences. This was followed by RNA isolation and semi-quantitative RT-PCR analysis for determining the expression of *AdTLP* gene in transgenic plants (Figure 9A). Actin amplification served as internal control. This analysis showed that the putative transgenic plants 7 and 4 were conferring highest and lowest level of expression respectively. The primary transgenic plants of #4 and #7 were selfed to obtain seeds for subsequent generations of the plants after reconfirmation using PCR and RT-PCR (Figure 9B).

### Rhizoctonia root rot assay

**Figure 10:**
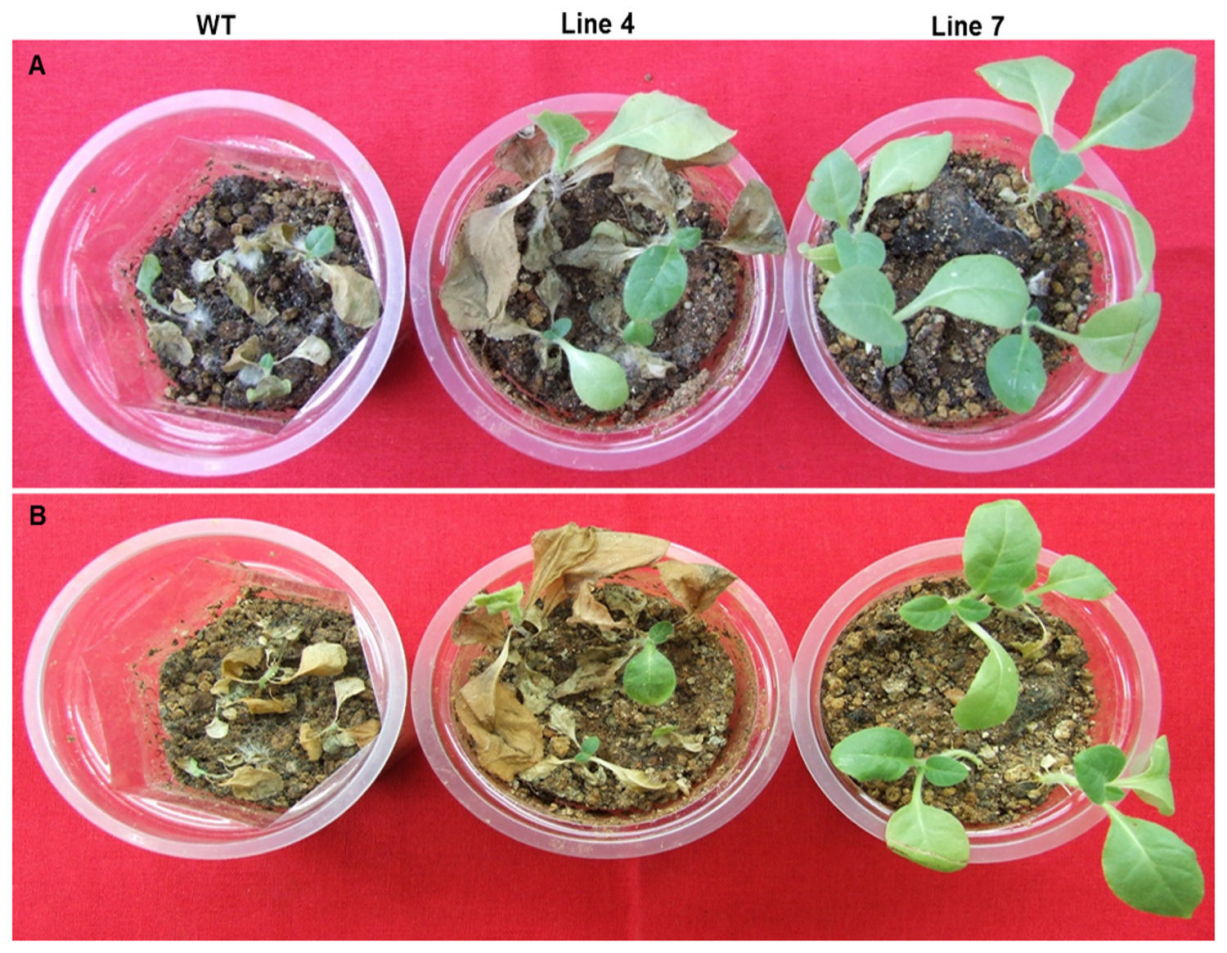
*Rhizoctonia* *solani* wilt bioassay with T_2_ transgenic and the non-transformed control. Fungal resistance was checked in control and transgenic plants using phytopathogenic fungus *R. solani*. Control plants were seriously affected whereas high expression line appeared completely healthy. Photographs were taken after 8 (A) and 10 days (B) post inoculation of fungus.

**Figure 11:**
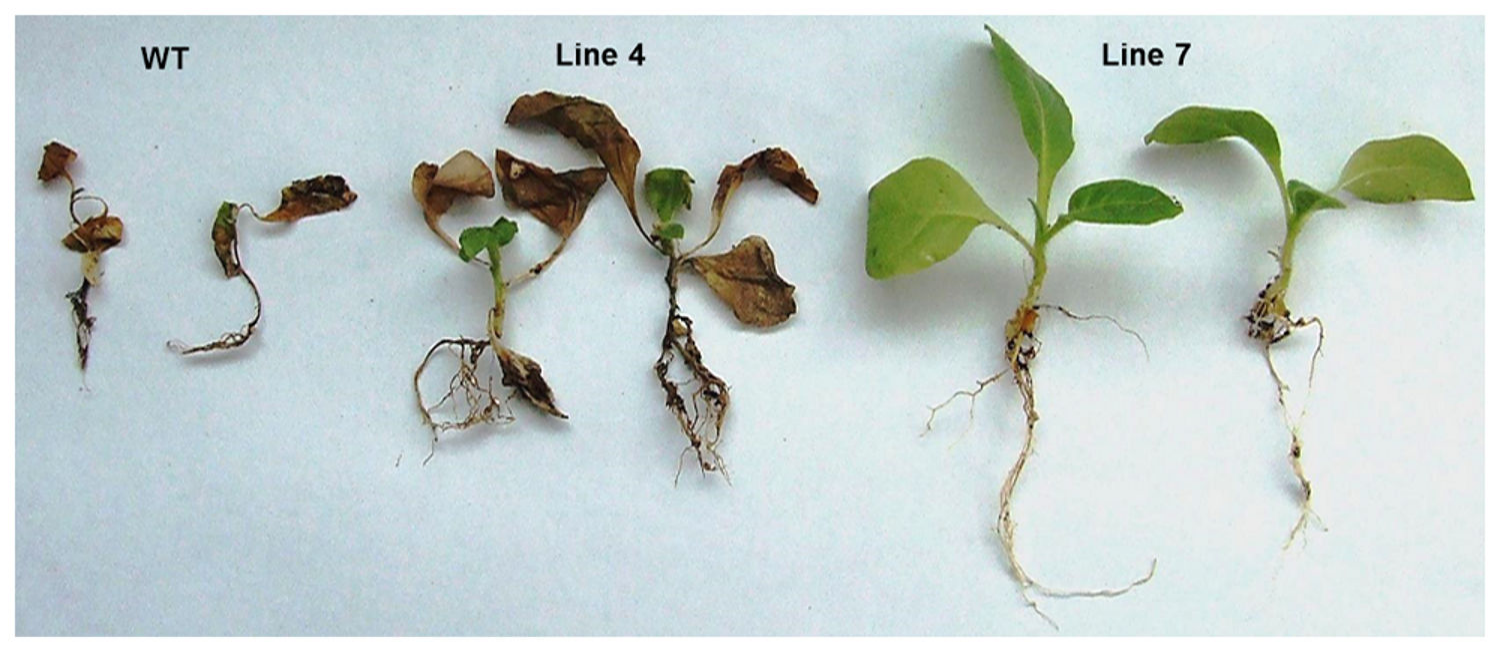
Condition of root, root and shoot junction and complete plant after 10 days of post inoculation of fungus. Two plants were taken from each line. Wild type plants became completely wilted. Infection symptoms also appeared in the roots of the low expression line plants whereas high expression line plants were completely healthy.

Antifungal resistance in transgenic plants was checked using the broad host range fungal pathogen, *Rhizoctonia solani* that causes seedling rot disease in several crop plants. T_2_ generation progeny of the low expression *AdTLP* plant, # 4 and the high expression plant, #7 were tested for fungal resistance along with control non-transformed plants in the assay. After 2 days of treatment using the sclerotia of the pathogen, fungal mycelia grew and covered the complete upper soil layer in the cups. The symptoms of infection started appearing in the wild type (WT) after 5 dpi. Fungal infection was severe on the control WT plants compared with the transgenic plantlets. After 10 dpi, WT plants became completely wilted and turned brownish black (Figure 10). Infection symptoms were also prominent in the progeny of the low expression plant 4, but the progeny plants of the high expression transgenic plant 7 were completely healthy. To check and compare the infection at root shoot junction, plants were uprooted from the cups and compared (Figure 11). Root growth of WT was completely retarded and the whole plant turned brownish indicating complete necrosis. The progeny plants of the transgenic #7 did not show any symptoms of infection whereas, some symptoms of wilting, necrosis and browning were observed in root and at root-shoot junction in the progeny of the plant 4. These observations showed that the high expression transgenic plant 7 exhibited enhanced levels of resistance against the root rot pathogen *R.solani* and *AdTLP* is a good candidate gene for imparting resistance against some fungal pathogens in crop plants. 

### Salinity and oxidative stress tolerance

**Figure 12:**
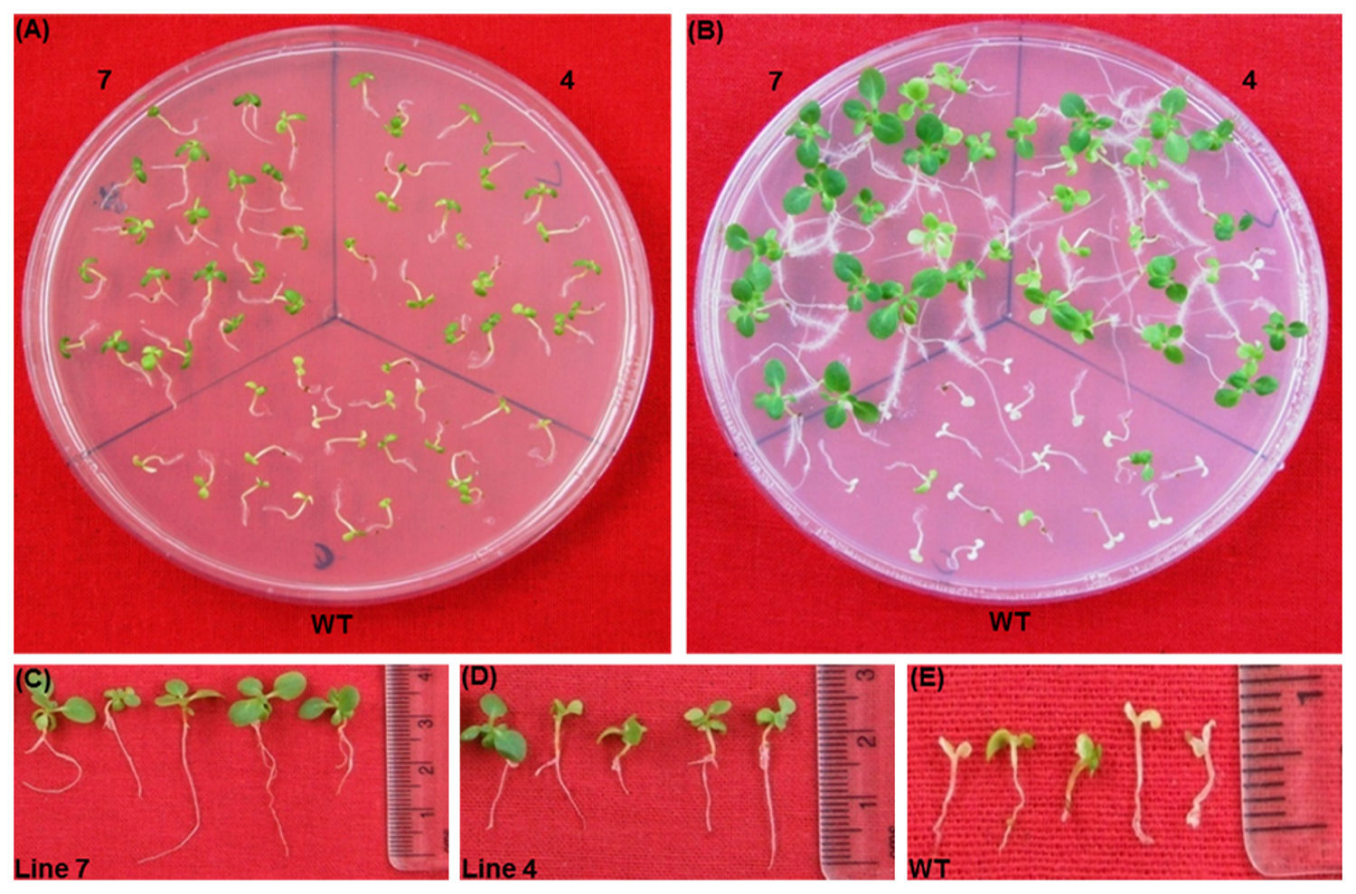
Seedlings assay with 200mM NaCl. Seedlings were transferred on 200mM NaCl medium for 14 days (A). Seedlings on recovery medium (B). Seedlings condition after 10 days of recovery period of line 7 (C), line 4 (D) and the wild type (E).

**Figure 13:**
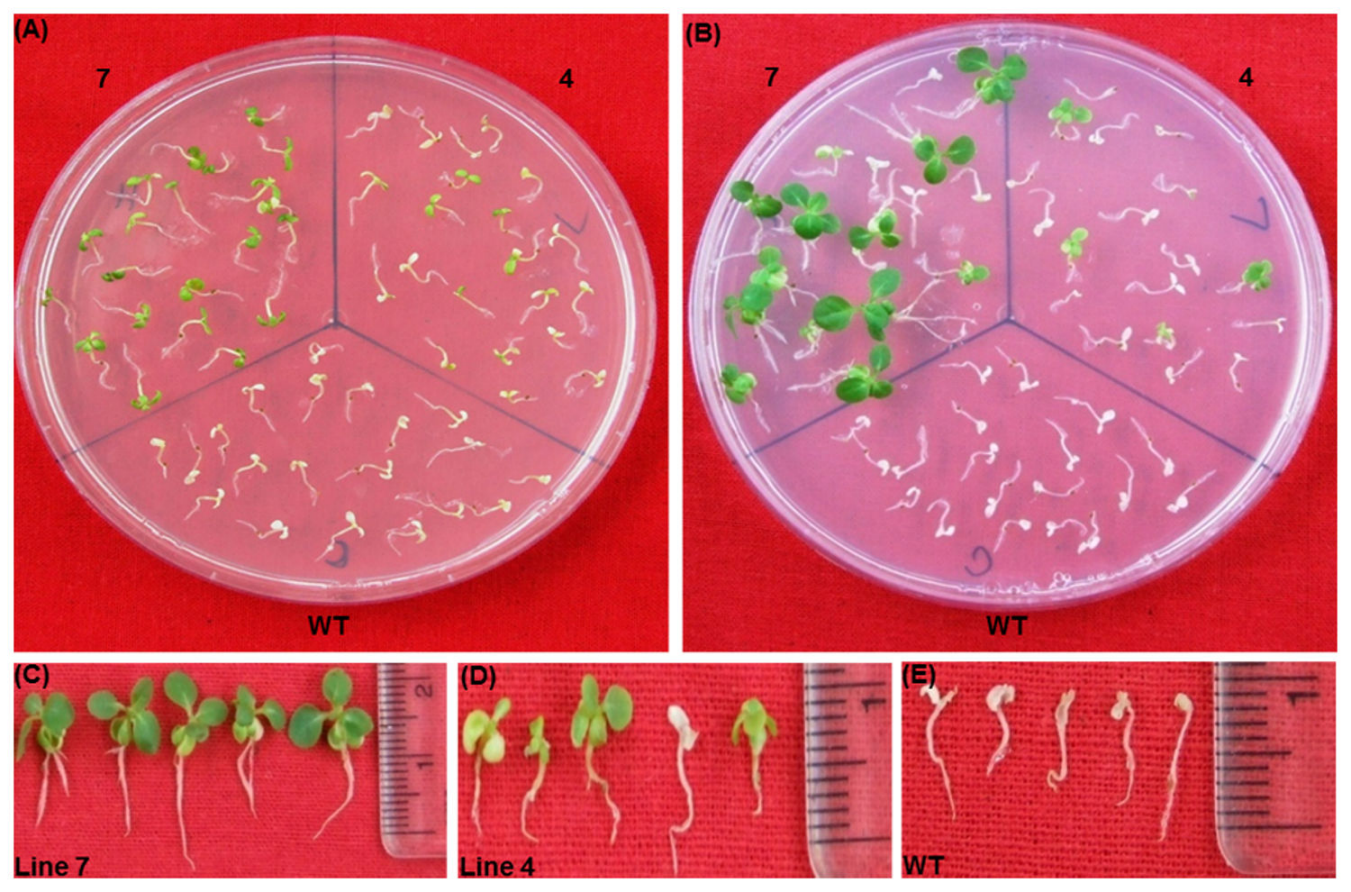
Seedlings assay with 300 mM Nacl. Seedlings were transferred on to 300mM NaCl medium for14 days (A). Seedlings on recovery medium (B). Seedlings condition after 10 days of recovery period of line 7 (C), line 4 (D) and the wild type (E).

**Figure 14:**
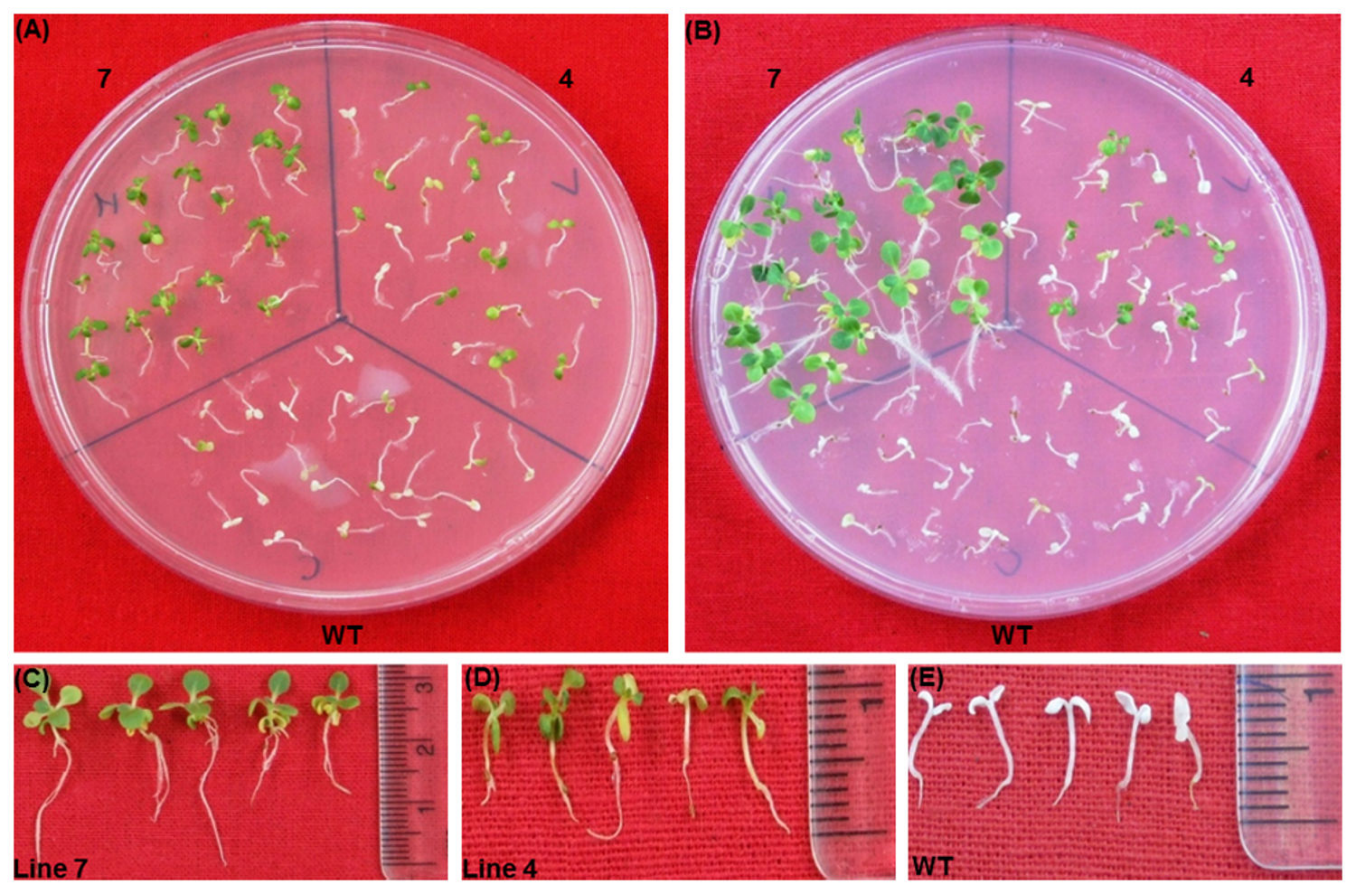
Seedlings assay with 2% H_2_O_2_. Seedlings were transferred on 2% H_2_O_2_ medium for 12 days (A). Seedlings on recovery medium (B). Seedlings condition after 10 days of recovery period of line 7 (C), line 4 (D) and wild type (E).

**Figure 15:**
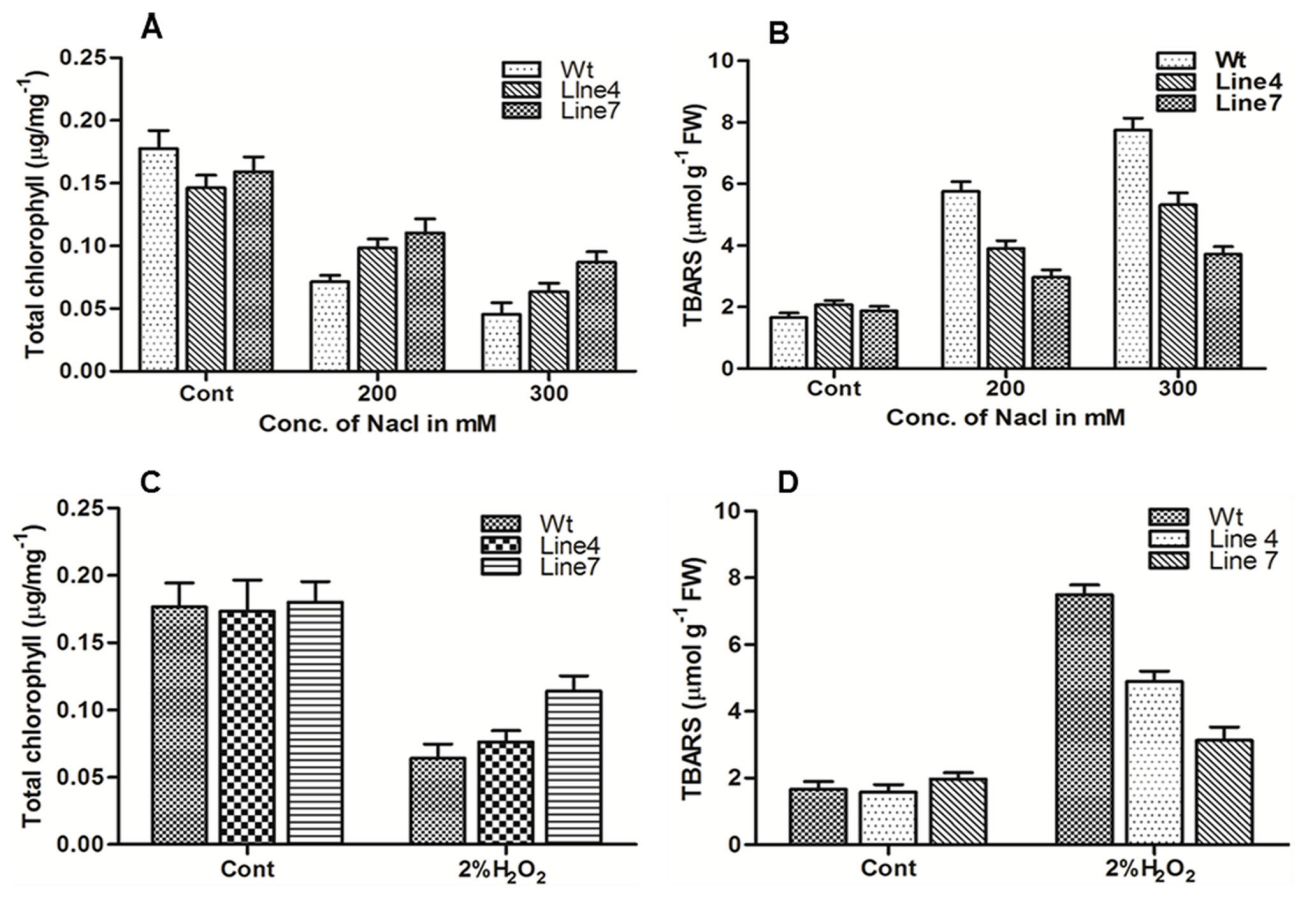
Total chlorophyll and TBARS measurement. Total chlorophyll and TBARS were measured in seedlings after 12d of NaCl treatment (A and B) and after 10d of H_2_O_2_ treatment (C and D). Note the significantly increased total chlorophyll content and reduced TRABS in the transgenic seedlings after stress treatments. Experiments were repeated three times and means ±SE were plotted (P < 0.05, n = 3).

To assess the tolerance of transgenic plants to salt stress, 10 days old seedlings of T_2_ generation of transgenics and WT were exposed to 100-300 mM NaCl in half MS-medium without organic nutrients. The 100 mM concentration of NaCl did not show any discernible differences between WT and transgenic as all seedlings were healthy and green even after 14 d exposure (data not shown). On 200 mM NaCl medium, both the lines of transgenic did not appear to be sensitive after 14 d of exposure, whereas better growth was observed in the progeny of the plant 7 compared to the progeny plants of plant 4 (Figure 12A). At the same time, chlorosis appeared with growth inhibition in WT seedlings. On a medium containing 300 mM NaCl, high level of bleaching was observed in the WT seedlings and almost 50% seedlings of plant 4 also got bleached out (Figure 13A). Only seedlings of the transgenic plant 7 showed better growth with very little chlorosis. To check the oxidative stress tolerance, seedlings were treated with 2% H_2_O_2_ in MSH medium without organic nutrients. Some chlorosis appeared in the newly grown leaves of transgenic seedlings, whereas WT seedlings became completely bleached out after 12 d exposure (Figure 14A). Total chlorophyll content and TBARS analysis showed that both salt and oxidative stress treated transgenic seedlings exhibited significantly higher chlorophyll (Figure 15A, C) content with low TBARS value (Figure 15B, D) compared to WT.

### Seedling recovery after stress treatment

To assess the recovery response of the WT and transgenic seedlings treated with various concentrations of sodium chloride and 2% H_2_O_2,_ they were transferred to NaCl and H_2_O_2_ free media. Since there were no significant differences between WT and transgenic seedlings grown on 100 mM NaCl medium, only the seedlings subjected 200-300 mM NaCl medium were transferred to the recovery medium. After 10 days, progeny seedlings of the transgenic plants 7 and 4 from 200 mM NaCl medium recovered completely and manifested near normal growth in comparison to WT seedlings, which were completely bleached out and did not recover (Figure 12B). From 300 mM NaCl medium, only the progeny seedlings of the plant 7 were only able to recover properly with true leaf formation and well-developed root system. The WT seedlings never displayed any signs of recovery (Figure 13B). Similarly among the seedlings from 2% H_2_O_2_ medium, only transgenic seedlings of the high expression plant 7 were able to recover well and give healthy appearance. Only very few seedlings from the plant 4 were able to recover, while the WT seedlings remained totally bleached (Figure 14B).

### Transcript level analysis of defense responsive genes in transgenic plants

**Figure 16:**
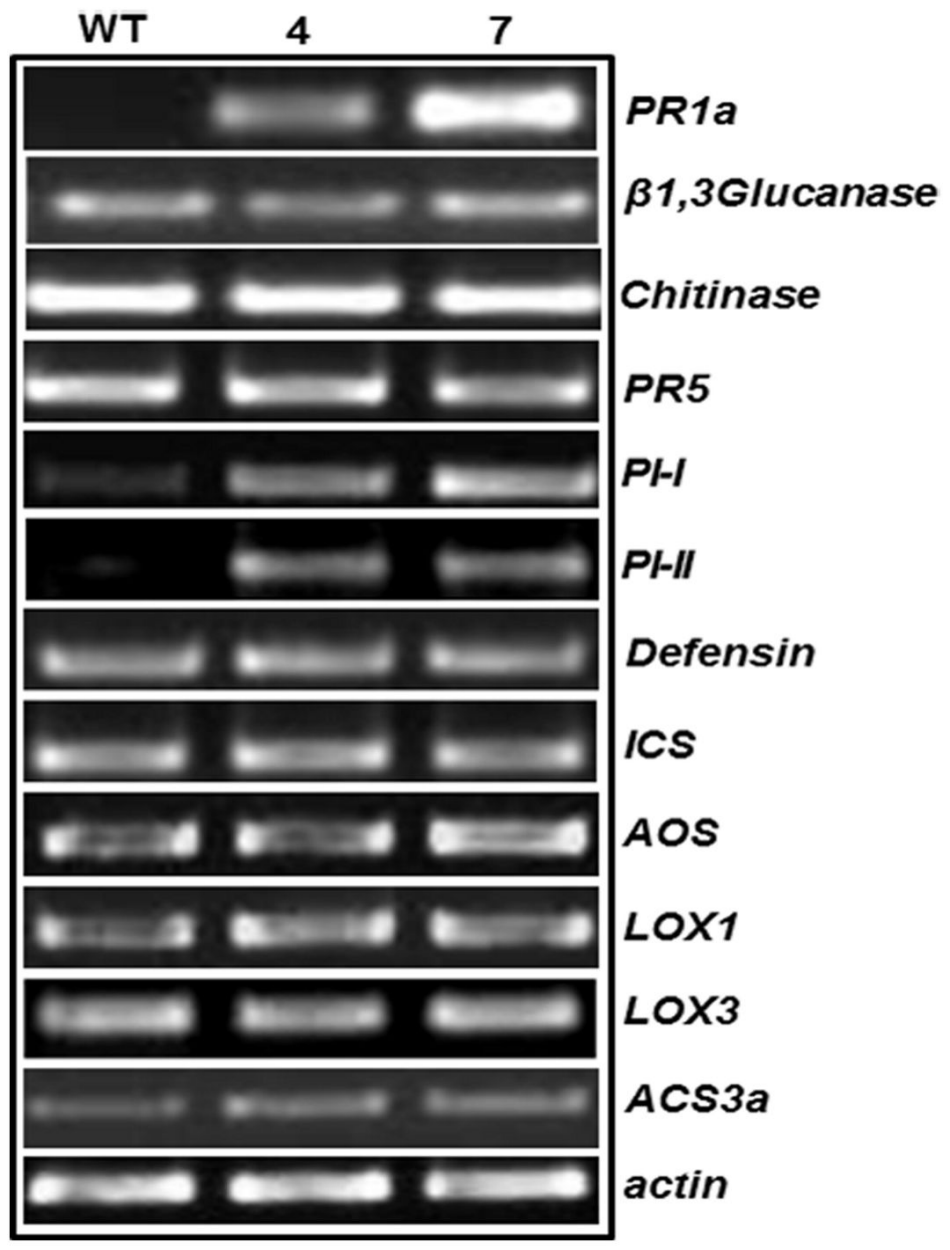
Transcript profile of defense responsive genes. Semi-quantitative RT-PCR was performed for transcript profiling of defense response genes in WT and transgenic plants. *PR*: Pathogenesis related proteins, *PI*: Protease inhibitor, Lox: Lipoxygenase, *AOS*: allene oxide synthase, *ICS*: isochorismate synthase, *ACS*: 1-aminocyclopropane-1-carboxylic acid synthase.

Transcript level of various defense related genes were analyzed in the transgenic plants using semi-quantitative RT-PCR (Figure 16). Constitutively higher transcript level of Pathogenesis related protein 1a (*PR-1a*), Protease inhibitor 1 (PI-1) and Protease inhibitor 2 (PI-II) were displayed by the transgenic plants compared to the WT plants. The transcript level of *ICS, Lox3* and *ACS3a*, which code for the key enzymes in SA, JA and Ethylene biosynthesis pathway respectively were unaffected. Transcript level of wound and Jasmonic acid responsive gene i.e. allene oxide synthase (AOS) and allene oxide cyclase (AOC) were almost similar to wild type. The Basic PR-5 (osmotin) and defensin genes that are synergistically regulated by ethylene and JA were also unaffected at the transcript level. 

## Discussion

Thaumatin-like proteins have been isolated and characterized from different plants and tissues. They are classified under the PR-5 proteins and are shown to be involved effectively in alleviating both biotic and abiotic stress tolerance [29–31]. In this study, a pathogen induced, full length 726 bp cDNA encoding a thaumatin-like protein from wild peanut, *Arachis diogoi* was amplified and cloned using partial AdDR-11 template as reference sequence that was identified as one of the upregulated genes in the wild peanut in pathogen challenge [22]. For our study, the gene was named as *AdTLP*. Sequence analysis revealed that AdTLP encodes a predicted protein of 241 amino acids which exhibited a 21 amino acid long N-terminal signal peptide with 25.01 kDa molecular weight and 4.71 theoretical pI. Phylogeneitc analysis revealed that the leguminous MtTLP and GmTLP showed closest similarity to AdTLP with 68% and 66% respectively. 

The induction of TLP upon pathogen treatment was reported in several plant-microbe interactions [3,32]. The transcript level of *AdTLP* got upregulated during *Phaeseoropsis personata* treatment and reached to the highest level after 72 hpi possibly showing its activity during the later stages of infection. The stress hormones involved in the signalling of biotic and abiotic stress responses like SA, JA and ABA had a positive induction effect on the *AdTLP* transcript levels suggesting a possible role of *AdTLP* in different stress responses with a significant cross talk. The recombinant TLP proteins like TaPR5-GFP [33] and CkTLP-GFP [34] were mainly identified as extracellular proteins during transient expression. Other TLPs like RlemTLP and CsTL1, despite being predicted as extracellular, were found predominantly localized to both periphery of plasma membrane and cytoplasm and involved in anti-fungal activities as well [35]. The localization analysis of recombinant AdTLP-GFP protein also showed extracellular localization and the observation was consistent with the prediction of secretory signal peptide of 21 amino acids. However, the AdTLP-GFP protein expression in subcellular structures, possibly ER regions, needs further investigation for confirmation. The presence of AdTLP-GFP protein in the nuclear boundaries could be due to the extension of ER from plasma membrane to nucleus or due to the overexpression of GFP fusion protein under 35S promoter. The antifungal activity of the recombinant AdTLP that was purified after induction in the *E.coli* prokaryotic system was checked using spore germination and plate assays with different filamentous fungal pathogens that attack various crop plants. During spore germination assay, various concentrations of protein were used and it was observed that 5µg/ mL of recombinant protein was sufficient to inhibit spore germination completely in the case of *F. oxysporum, F. solani* and *B. cinerea* for which the calculated IC_50_ values were less than 1µg/ mL. These values were significantly low compared to a legume TLPs [36,37] as well as osmotins and TLPs from other plants [12,35]. Apart from growth inhibitory activity, the AdTLP also induced morphogenic changes like hyperbranching in the mycelium of fungal pathogen, *B.cinerea* in a way similar to the legume defensins [24] showing it to be a potent antifungal protein. The IC_50_ value of *R. solani* was 38µg/ mL. These results suggested better efficiency antimicrobial activity and broad spectrum of AdTLP protein against fungal infection.

β-1, 3 glucan is a common component of fungal cell wall. Some TLPs have been reported to display endo-β-1, 3 glucanase activity [14,38], which could be one of the possible mechanisms for their antifungal activity. However, when the recombinant AdTLP protein was incubated with laminarin as substrate, no detectable absorbance was observed even after 72 h indicating that this protein did not possess glucanase activity.

It was reported that transgenic tobacco plants overexpressing rice thaumatin-like protein showed enhanced resistance to *Alternaria alternate* [39]. Similarly, transgenic tobacco plants overexpressing thaumatin-like protein gene from cotton fibre showed enhanced resistance against *Verticillium dahliae* [40]. For this reason, the antifungal activity of AdTLP in tobacco transgenic plants was checked against a wide range of plant pathogenic fungi. *Rhizoctonia solani*, which infects the roots and lower parts of the stem, is a serious pathogen affecting a large number of plant species [41] and one of the test pathogens used in the present study. Our results showed that the progeny plants of the primary transgenic plant 7 exhibited superior tolerance with no signs of any infection even after 10 dpi, whereas infection symptoms developed late in progeny of the plant 4. WT plants were seriously affected and damaged completely after infection. Difference in the level of resistance between plants 7 and 4 could be correlated with expression level of *AdTLP* gene. The experiment showed *in vivo* effectiveness of AdTLP protein during fungal attack.

Earlier studies suggested the involvement of TLPs in enhancing tolerance to various abiotic stresses in plants with heterologous/ constitutive/ enhanced expression of TLPs. For instance, the transcript level of wheat TLP increased remarkably after the treatment with ABA and elicitors [42]. Similarly, Wang et al. [34] showed that the expression levels of CkTLP from *Cynanchum komarovii* seeds got upregulated during ABA, NaCl, drought, MeJA and SA treatments indicating that CkTLP might play an important role in response to abiotic stresses also. Transgenic approaches also confirmed their role in enhanced tolerance in transgenic plants expressing TLPs. Rajam et al. [43] reported higher percentage of transgenic seed germination and survival during salt and drought stress conditions in transgenic tobacco plants expressing *Thaumatococcus daniellii* TLP. A cotton fibre TLP gene, as discussed above, was also involved in tolerance during salt, drought and oxidative stress [40].

The present set of tobacco transgenic plants expressing AdTLP also exhibited tolerance to sodium chloride (osmotic/oxidative stress) and hydrogen peroxide (oxidative stress) when checked *in vitro*. Treatment with 200 and 300mM NaCl showed that transgenic plants 7 and 4 tolerated 200mM salt stress treatment, whereas the high expression plant 7 was better capable of tolerating 300 mM treatment.

Accumulation of reactive oxygen species occurs during the salt stress condition causing oxidative damage to the membrane proteins, lipids and nucleic acid [44]. It leads to the chloroplast damage and depletion in the chlorophyll levels. Membrane damage results in higher TBARS levels in plants under stress. In case of *AdTLP*, the higher chlorophyll contents and lower TBARS suggested that the transgenic lines were more salt tolerant compared to WT with significant lower membrane damage. The transgenic tobacco plants overexpressing *GbTLP* were able to tolerate H_2_O_2_ stress condition upto 2% and maintain higher chlorophyll content [40]. Hence, direct effect of oxidative stress was also checked by transferring WT and transgenic seedlings on 2% H_2_O_2_ media. Despite chlorosis that appeared after 12 days of treatment, transgenic seedlings showed higher chlorophyll content with low TBARS value compared to WT and indicated their higher tolerance level with less chloroplast damage and better membrane integrity. Based on the recovery results, it appears that the constitutive expression of *AdTLP* can partially reverse the stress induced growth inhibition.

Recent studies have shown that PR5 proteins are not the eventual component of signal transduction cascade. They might indirectly contribute to other defense regulatory mechanisms in plants. A TLP protein from *Prunus domestica* was involved in activating the genes of phenylpropanoid and phytoalexin pathways in Arabidopsis along with antifungal activity [45]. Similarly CsTLP from *Camellia sinensis* was assosiated with the activation of *LOX* and phenylpropanoid pathway in potato [46]. In addition, PR5 overexpression has been found to increase H^+^-ATPase activity, improved seed germination and involved during senescence in other species [47–49]. In order to verify the involvement of AdTLP in defense responses, we studied the transcript levels of various defense related genes in WT and transgenic tobacco plants using semi-quantitative RT-PCR. The transgenic lines showed higher transcript levels of *PR1a*, *PI-I* and *PI-II* genes compared to WT. *PI-I* and *PI-II* encode protease inhibitors and are believed to be the components of insect defense mechanism in plants. Various TLPs have been reported to be induced during wounding and insect attack [50–52]. Microarray analysis of maize seedling during insect attack had shown differential expression of thaumatin-like protein genes along with other genes [53]. Significant increase in TLP expression occurred in poplar phloem during wound treatment and the protein presence was confirmed by immunolocalization studies [54]. The exact reason of TLPs involvement during such stress events has not yet been clearly elucidated. Though α-amylase and protease inhibitor activities are major modes of action of plant resistance proteins to insect attack, these possibilities have been ruled out for PR5 proteins [55]. Our observation during this study indicated that AdTLP protein might play some role in plant defense during insect attack by inducing *PI-I* and *PI-II* genes in transgenic lines. However, due to lack of full understanding about the mode of action of TLPs [5,56], it is difficult at present to predict the exact mechanism behind the induction of other genes in *AdTLP* transgenic plants.

 There are several examples suggesting that TLPs can interact with a variety of ligands and proteins including actin [57], cytokinin [58] and viral proteins [59]. The reason behind their involvement in various functions can be understood based on their ability to adopt functional diversification despite their conserved nature. Probably these became possible in TLPs through multiple functions due to the mutations in appropriate amino acid residues, which might have occurred during the course of evolution [5]. Not only TLPs but also other PR proteins like PR3 [60] and PR14 [61] have also been reported in diversified functions. Finally, our observations suggest that further in depth study is needed for *AdTLP* to explain the mode of action and their involvement in induction of other genes, which ultimately could help unravel a better picture in understanding the functional aspects of TLPs.

## Conclusion

In summary, we have amplified full length *AdTLP* gene from *Arachis diogoi* (a wild species related to the economically important legume crop peanut), which was expressed in its interaction with the pathogen, *Phaeoisariopsis personata*. The *in vivo* and *in vitro* activities of a thaumatin-like protein have been analyzed. The protein has imparted significant resistance against fungal pathogens, salt and oxidative stress. Apart from this, the transgenic plants also displayed higher transcript level of *PR1a*, *PI-I* and *PI-II* gene compared to WT. Taken together, these observations suggest that the *AdTLP* can be a good candidate gene for deployment in transgenic plants for enhancing their tolerance against various biotic and abiotic stresses.

## Supporting Information

Figure S1 **12% SDS-PAGE analysis showing protein profiles.** (M) Protein marker, (UI & I) uninduced and induced proteins respectively.
(TIF)

Table S1 **Sequences of gene specific primers used in semi-quantitative RT-PCR for amplification of defence related gene transcripts.**
(DOCX)
