# Supplementary figures and images for: Characterization of a Pathogen Induced Thaumatin-Like Protein Gene AdTLP from Arachis diogoi, a Wild Peanut
Source: PLoS One. 2013 Dec 19;8(12):e83963. doi: 10.1371/journal.pone.0083963 (PMC3868660; doi:10.1371/journal.pone.0083963)

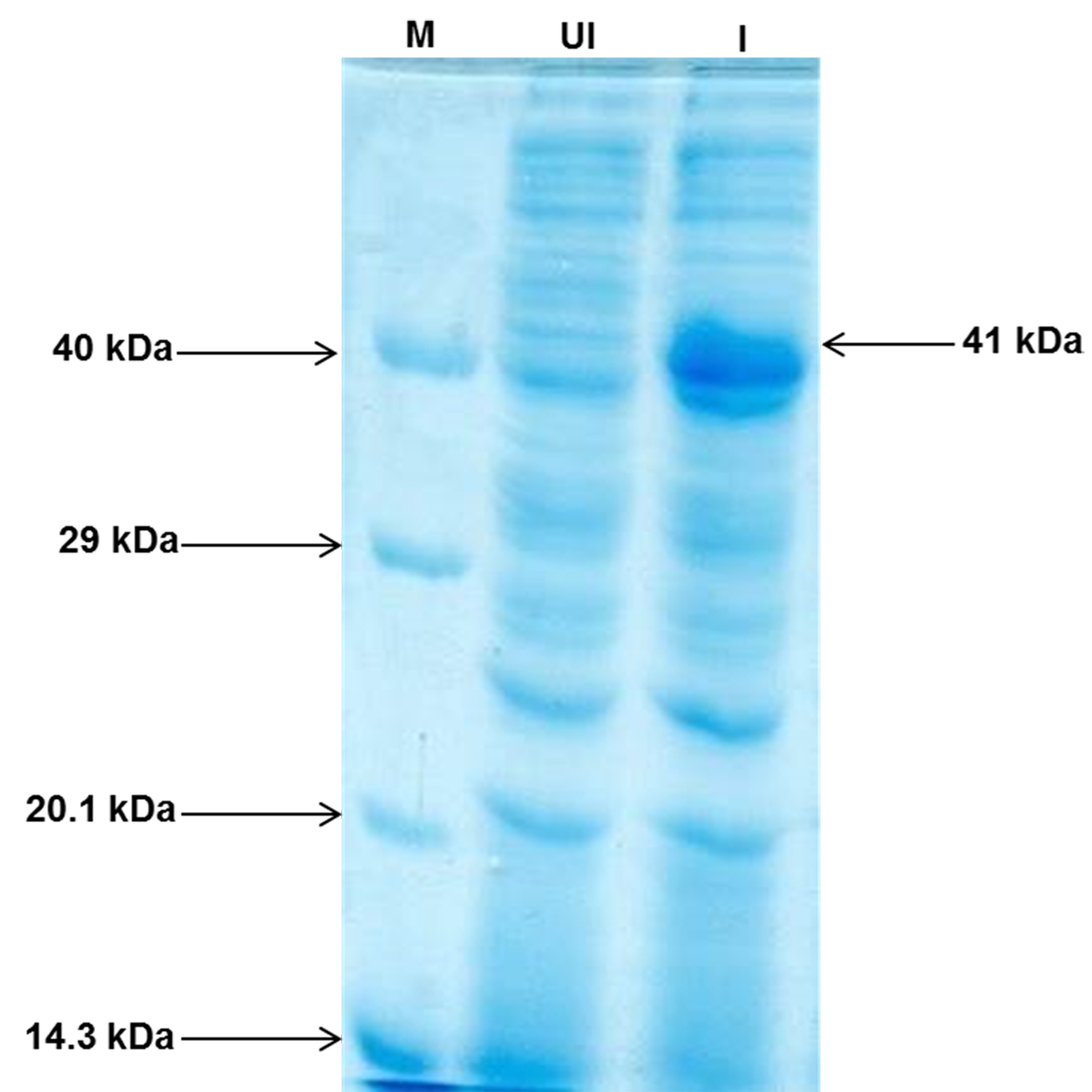

Supplement: Figure S1 — 12% SDS-PAGE analysis showing protein profiles. (M) Protein marker, (UI & I) uninduced and induced proteins respectively. (TIF) [file pone.0083963.s001.tif]
