# Supplementary material for: Characterization of a Pathogen Induced Thaumatin-Like Protein Gene AdTLP from Arachis diogoi, a Wild Peanut
Source: PLoS One. 2013 Dec 19;8(12):e83963. doi: 10.1371/journal.pone.0083963 (PMC3868660; doi:10.1371/journal.pone.0083963)
Supplement: Table S1 — Sequences of gene specific primers used in semi-quantitative RT-PCR for amplification of defence related gene transcripts. (DOCX) [file pone.0083963.s002.docx]

**Table S1.** Sequences of gene-specific primers used in RT-PCR for amplification of defence related gene transcripts. See text for descriptions.

| **Oligo name** | **Forward** | **Reverse** |
| --- | --- | --- |
| *PR1a* | CTTCTTGTCTCTACACTTCTC | GCAAGAGACAACATATCCTC |
| *Chitinase* | CTGAAGAATAGGAACGACGGTAG | ATACCTCCTGTAGTATCCAATTCG |
| *Glucanase* | ATGGCTTTATGCATTAAAAATGGC | AGCATTGAAGACATTTGTTTCTGG |
| *Defensin* | GAGGCGAGAACTTGTGAGTC | AAGCCGAAACCATTATTCATAAC |
| *PR5* | CTTGAGATCTTCTTTTGTTTTCTTC | ACTTCCAGGCATTTCCAAGGGAAA |
| *PI-I* | ATGGTGAAGTTTGCTCACGT | AATCCCTTAGCCAACCTGG |
| *PI-II* | GTTAGTTTCGTCGCTCATCT | CTGCGTTACAACAGTTGATG |
| *ICS* | TGCATATCAGTTCTGTTTGCAAC | CCAGCATACATTCCTCGGTCA |
| *AOS* | CTTGGTCTTCCGAAGGTTC | GACGTCGATATCCAACGTG |
| *ACS3a* | ATAGTTATGAGTGGAGGAGC | CCGTGTCTTTTCCCTAGTCT |
| *LOX1* | CACTTCCTACTGATCTCATC | CTCATCGACATTCATCTGCA |
| *LOX3* | AATGACAGAGAACTCCAAGC | TAGAACGCTTCGACAATCTC |
